# Genomic insights into population structure and adaptive variation of *Pimelodus yum*a and *Pimelodus grosskopfii* in the Magdalena-Cauca Basin

**DOI:** 10.1371/journal.pone.0351301

**Published:** 2026-06-05

**Authors:** Hayler Edu Ibarra Arcila, Juan Aicardo Segura Caro, Edna Judith Márquez, Jose Gregorio Martinez

**Affiliations:** 1 Grupo de Investigación Biociencias, Maestría en Biotecnología y Bioeconomía, Facultad de Ciencias de la Salud, Institución Universitaria Colegio Mayor de Antioquia, Medellín, Antioquia, Colombia; 2 Grupo de Investigación en Biotecnología Animal, Laboratorio de Biología Molecular y Celular, Escuela de Biociencias, Facultad de Ciencias, Universidad Nacional de Colombia, Sede Medellín, Medellín, Colombia; DePaul University, UNITED STATES OF AMERICA

## Abstract

The biodiversity of the Magdalena–Cauca Basin, Colombia’s main fluvial system, is under severe threat from anthropogenic activities, imperiling endemic fish species such as *Pimelodus yuma* and *Pimelodus grosskopfii*. Using a population genomic approach based on single nucleotide polymorphisms (SNPs), we analyzed 64 individuals of *P. grosskopfii* and 57 individuals of *P. yuma* collected across ~1,600 km of the Magdalena–Cauca Basin. We identified two coexisting genetic stocks in both species, maintained by restricted gene flow that is associated with adaptive divergence rather than geographic distribution. Selection pressures, likely linked to the basin’s bimodal hydrological regime, were detected as major drivers of genetic structure. Historical demographic reconstruction analyses indicate that stock 1 in both *P. grosskopfii* and *P.* *yuma* was established in the basin during the Late Miocene–Pliocene (~4.5–5.5 MYA), with *P. grosskopfii* exhibiting an early expansion followed by long-term stability up to the present, while *P. yuma* maintained a stable population size until a recent decline. Stock 2 in both species was established during the Early Pleistocene (~1.7–2.5 MYA), followed by expansion and stability in *P. grosskopfii*, and a stable population size followed by a contraction–recovery–expansion dynamic in *P. yuma*, suggesting long-term persistence of neutral/adaptive processes shaping these stocks. Each stock should be considered a Management and Adaptive Unit, highlighting the need for targeted conservation actions and broader strategies to ensure their persistence under ongoing environmental and anthropogenic pressures in the Magdalena–Cauca Basin.

## Introduction

The Magdalena–Cauca basin, spanning approximately 273,000 km², is Colombia’s most important region in terms of population density (78% of the national population) and economic contribution. It is formed by the Magdalena River (1,538 km) the Cauca River (1,350 km), San Jorge River and Nechi River, together constituting a fluvial system with floodplains extending over 2 million hectares [1]. Of these, 326,000 hectares are permanent wetlands (ciénagas), varying in size from 1 to 11,000 hectares, which serve as critical spawning and refuge habitats for the basin’s ichthyofauna [2].

The Magdalena River is ecologically more diverse, more extensive, and more environmentally impacted by anthropogenic activities. For instance, the basin currently faces a severe environmental crisis due to overexploitation of resources, habitat degradation, dam construction affecting migration, and pollution from hydrocarbon industries, including heavy metals like mercury, lead, and cadmium [3,4]. In recent decades, the Magdalena has experienced one of the highest erosion rates globally (710 t/km²/year), with deforestation exceeding 70% and wetland loss surpassing 80%, directly affecting critical habitats for fish reproduction, recruitment, and refuge [5].

The basin hosts remarkable freshwater fish diversity, with 237 recorded species, accounting for approximately 14.5% of Colombia’s freshwater fish diversity [5]. Among them, 158 species are endemic, including members of the genus *Pimelodus* [5].

The catfishes of the genus *Pimelodus* (Siluriformes: Pimelodidae) are widely distributed across Neotropical freshwater ecosystems in South America, where they constitute a key resource for both artisanal and commercial fisheries. Two species, *Pimelodus* *grosskopfii* and *Pimelodus yuma*, are endemic to the Magdalena–Cauca basin in Colombia and exhibit a differential altitudinal distribution. *P. yuma* is found in the lower sections of the Magdalena, Cauca, and Sinú River drainages (~26–587 m a.s.l.), whereas *P. grosskopfii* occupies a broader elevation range along the Magdalena and Cauca rivers (~26–1036 m a.s.l.). Consequently, both species coexist in the lower parts of the basin, creating a natural zone of sympatry [6]. Both species have been identified as medium-distance potamodromous migrators, covering distances of approximately 100–500 km within the Cauca River [7,8]. While *P. grosskopfii* is classified as Critically Endangered (CR) by the IUCN, *P. yuma* remains designated as Data Deficient (DD) due to a lack of the scientific information required for a formal risk assessment. Between 1975 and 2015, fishery yields in the basin declined by 56.25% for several flagship species [9], leading to a shift in fishing pressure toward these two catfish species, which has significantly intensified in the past decade [10–13].

One major consequence has been the erosion of their genetic diversity, potentially compromising their adaptive capacity and long-term survival. For instance, two studies published in 2021 identified elevated inbreeding coefficients in populations from the Cauca sub-basin, highlighting a substantial risk of extinction [7,8]. Besides the overfishing, the degradation of Magdalena-Cauca basin caused by factors like economic, environmental and demographic, contributes to the observed decline in the number of catches of these fishes [7,8].

The Magdalena-Cauca River exhibits a marked bimodal flood regime, featuring two distinct high-water periods associated with rainy seasons from April to June and October to December, and two low-water periods linked to drought seasons from January to March and July to August [14]. Accordingly, most of potamodromous fish species in the Magdalena–Cauca basin exhibits two reproductive picks seasons annually: the *subienda* during the first half of the year and the *mitaca* in the second half, each aligned with one of the two flood pulses. These upstream migrations last approximately 30–45 days. Following the onset of rainfall, individuals spawn and either remain in the river (residents) or initiate downstream migrations (*bajanza*) to return to floodplain lakes, which serve as nursery, refuge, and feeding habitats [3,15]. Nevertheless, migrations in these potamodromous fishes are driven not only by reproduction but also by feeding—a fundamental activity that occurs during both high- and low-water periods across species, with variations in their abundance and co-occurrence, depending on their dietary habits (Atencio-García, 2025, pers. comm.).

Regarding the reproductive dynamics of both species, it is known that they respond to both annual reproductive seasons of the Magdalena–Cauca basin. After migrating upstream and spawning, fertilized eggs drift downstream, hatching along the way and allowing larvae to reach floodplain lakes where they feed and grow until the next reproductive cycle begins [15]. Although both species reproduce twice per year (once per high-water pulse), it remains unclear whether the same individuals undergo gonadal maturation for both reproductive events or whether distinct populations or genetic stocks exist within each species, each responding exclusively (asynchronously) to a single reproductive event. This phenomenon is known as reproductive timing, potentially driven by distinct migratory ecotypes [16,17]. Captive reproductive biology studies suggest annual—not biannual—cycles of gonadal maturation and reproduction within individuals [18].

Previous studies using microsatellite loci have revealed that populations of *P. yuma* and *P. grosskopfii* comprise two coexisting genetic stocks, each exhibiting gene flow in the middle and lower sections of the Cauca River [7,8,19]. This structure was hypothesized to result from a spatial or temporal Wahlund effect [7,8]. A similar two-stock pattern was recently identified with microsatellites in *Cyphocharax magdalenae* [20], a Characiform endemic to the Magdalena–Cauca basin [4]. In that case, each stock was associated with environmental variables of the rainy and dry seasons, suggesting that ecological adaptation, rather than geographic barriers, may shape genetic structure by restricting gene flow in the Cauca sub-basin [20].

While this structured pattern has been repeatedly observed in various species within the Cauca River, the behavior of *P. yuma* and *P. grosskopfii* populations in the Magdalena River remains unclear. Indeed, genetic studies have revealed microevolutionary effects of such disturbances on migratory catfish species in Cauca, such as *Pseudoplatystoma magdaleniatum*, *Prochilodus magdalenae* in terms of shifts in allele frequencies and allelic loss [21].

Although microsatellites have been effective in inferring neutral variation in the Cauca—evidencing genetic diversity and population structure—such markers need to be complemented with the detection of adaptive variation, which is essential for the conservation of species in natural ecosystems [22], especially in complex systems like the Magdalena River, where both neutral and adaptive information remain unavailable for these and many other species.

Thus, in this study, we applied a genomic approach using single nucleotide polymorphisms (SNPs), which can integrate both neutral and adaptive genetic information. This approach facilitates management decisions, supports the establishment of foundational knowledge about population modeling in natural systems [22,23], and enables a more complete understanding of the evolutionary history of species and the forces shaping them.

Accordingly, the expectations of this study were: (i) to detect strong geographic structure with more defined patterns than those previously observed within the Cauca [7,8], given that this is the first time individuals from both the Cauca River (460 km) and the Magdalena River (1,200 km) have been sampled for both species, yielding over 1,600 km of linear sampling across the entire main basin; and (ii) to determine whether the sole force shaping this structure and the patterns of genetic diversity is the neutral variation (i.e., genetic drift).

## Materials and methods

### Sampling, DNA extraction and sequencing

**Fig 1:**
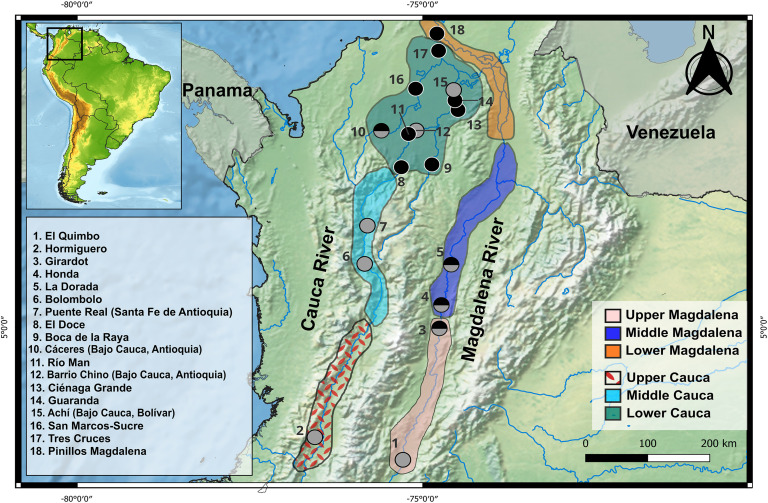
Sampling sites along ~1600 linear kilometers of the upper, middle, and lower sections of the Cauca and Magdalena rivers, including floodplain lakes and wetlands. Gray circles represent *Pimelodus grosskopfii* and black circles represent *Pimelodus yuma*. Bicolored circles indicate sites where individuals of both species were sampled. River sections are color-coded as follows: red for Upper Cauca, light blue for Middle Cauca, green for Lower Cauca, pink for Upper Magdalena, indigo blue for Middle Magdalena, and orange for Lower Magdalena. River network data were obtained from *Rivers in Colombia* (World Bank Data Catalog; public domain), licensed under Creative Commons Attribution 4.0 (CC BY 4.0), available at https://wbwaterdata.org/dataset/rivers-colombia. Background raster data were obtained from Natural Earth (public domain), available at http://www.naturalearthdata.com/. The map was constructed using these data sources in the R environment (https://www.r-project.org/).

Sixty-four muscle tissue samples of *P. grosskopfii* and fifty-seven of *P. yum*a were collected between 2014 and 2021 and provided by Integral S.A., through two scientific cooperation agreements (19th September 2013; Grant CT-2013–002443), by Instituto Humboldt, and less than 10% obtained under collection permit ANLA 1467 de 2019. All samples were collected at 24 sites along ~ 460 km and ~ 1200 km in the mainstream and floodplain lakes of the upper, middle and lower sections of the Colombian Cauca River and Magdalena River, respectively (Fig 1; S1 Table), during the dry season, a period that facilitates capture by traditional fishing gear. DNA was extracted from ethanol-preserved tissues by using the GeneJET Genomic DNA Purification Kit (Thermo Scientific). DNA quality was evaluated by electrophoresis on 0.8% agarose gels and EZ-vision® staining (AMRESCO). DNA was quantified with a NanoDrop 2000 spectrophotometer (Thermo Scientific) and diluted to a final concentration ranging from 30 to 50 ng/uL.

Both RAD library preparation and sequencing were carried out by the National Center of Genomic Sequencing (Colombia) in collaboration with Floragenex Inc. (USA), following their internal protocols (see https://floragenex.com for more details). At least 0.5 μg of high-molecular-weight DNA per individual was used for RAD library preparation. Genomic DNA was digested using the 6-bp restriction enzyme *Pst*I, followed by fragmentation via sonication. Libraries were constructed through adapter ligation, PCR amplification, and size selection targeting fragments within a range of approximately 200–300 bp, following proprietary internal protocols for RAD-seq library preparation of Floragenex. Sequencing was performed on a single lane of an Illumina HiSeq 2000 machine, generating single end reads of ~125 bp. This sequencing configuration was expected to produce approximately 80 million reads per lane, yielding at least 0.5–1 million reads per individual and an average sequencing depth per locus of approximately 10–30 × , depending on library complexity and quality.

It is important to clarify that all biological material used in this study consisted exclusively of tissue samples provided by third parties through institutional agreements described above. In addition, DNA samples and tissue remnants are available in the Molecular Biology Laboratory at the Universidad Nacional de Colombia, Medellín campus (see S1 Table).

### Genomic data processing

After sequencing, the genomic data quality was analyzed in FASTQC (Bioinformatics Group at the Babraham Institute, UK) [24]. The samples were demultiplexed using cutadapt [25], and SNPs were extracted using DiscoSnpRad [26] with default parameters except that presence indels and up to five SNPs per locus were permitted for construction of the loci. The resulting Variant Call Format (VCF) was quality filtered considering a Phred score  >  20, it was removed variants detected in 20% and less populations, set minimal read depth of seven per allele and finally, filtered to eliminate potential paralogues (SNP rank  <  0.4) [23]. This SNP rank, implemented in DiscoSnpRad [26], is a score ranging from 0 to 1 and reflects the confidence that a variant corresponds to a true bi-allelic polymorphism rather than arising from sequencing errors or collapsed paralogous regions. The SNP rank is computed from local graph properties in the de Bruijn graph, including coverage balance between alleles and the structural consistency of variant-associated bubbles. Low SNP rank values are typically associated with variants showing abnormal coverage patterns or complex graph structures, which are indicative of potential paralogous sequences or repetitive regions. Therefore, we applied a conservative threshold and removed all variants with SNP rank < 0.4, retaining only high-confidence SNPs for downstream analyses.

To generate the final genetic dataset used for population genetic downstream analyses (e.g. SNPs genotype matrix in VCF, STRUCTURE, GENPOP or ARLEQUIN format), we used the component populations.pl implemented in the software STACKS v.1.2 [27] using as inputfile the resulting filtered VCF above generated by DiscoSnpRad. The filtering parameters in STACKS included that each individual was considered a population in the map file, then the minimum number of populations (-p) that a locus must be present in to process it was set to 90% of the total sample (10% of missing data). Other filtering values for “-p”, tolerating ∼0% and ∼ 5% of missing data in the final dataset were also explored according to Martínez et al (2022) [28], however, these last two datasets showed less statistical power to discriminate samples (in terms of explained variance) and were then discarded for downstream analysis. Likewise, in STACKS, the minimum percentage of individuals in a population required to process a locus for that population was set to 100% (−r = 1), as well as a minor allele frequency (MAF) of 0.01 and retained only one SNP per locus. All other parameters for the analysis were kept as default settings.

Considering that statistical analysis that seek to infer gene flow, structure and diversity indices are based on the principles of neutral variation, we decided to examine the existence of outlier loci to remove them from the final dataset. For this, an analysis was performed with BAYESCAN v.2.1 software [29], which identifies outliers loci using differences in allele frequencies between populations with the best efficiency when compared with alternative programs to detect outlier loci [30]. For our analysis, parameters for the BAYESCAN analyses included 10:1 prior odds for the neutral model, and 20 pilot runs consisting of 5,000 iterations each, followed by 250,000 iterations with a burn-in length of 50,000 iterations. The loci were ranked according to their estimated posterior probability, being classified as putative outlier when ≥0.95 [31].

Finally, after removing outlier loci, the neutral dataset was evaluated for linkage disequilibrium using the standardized index of association (rbarD) implemented in the poppr package [32]. This statistic measures the overall non-random association of alleles across loci (i.e., multilocus linkage disequilibrium), allowing assessment of the degree of non-independence among markers. Unlike pairwise measures such as the squared allelic correlation (R²), which quantify linkage between specific pairs of loci, rbarD provides a global estimate of linkage disequilibrium (LD) across the dataset [32]. In this study, a threshold of rbarD > 0.2 was used as a conservative criterion to identify potential non-independence among loci and reduce redundancy in the SNP matrix, following Escobar et al. (2024) [23].

The RADseq raw demultiplexed dataset has been submitted to NCBI as Bioproject PRJNA1217604.

### Population structure analysis

For the purposes of geographic population analysis, samples of both *P. grosskopfii* and *P. yuma* were grouped according to natural longitudinal sections commonly recognized for the Cauca River (Upper Cauca, Middle Cauca, and Lower Cauca) and the Magdalena River (Upper Magdalena, Middle Magdalena, and Lower Magdalena) [33,34]. However, since the Lower Cauca and Lower Magdalena regions are geographically and bioecologically adjacent and given the reduced individual sample sizes in these areas for this study, they were combined to ensure statistical robustness, being collectively designated as samples from the Lower Cauca-Magdalena Basin, hereafter referred to as ‘Lower Cauca’ for graphical purposes. In the end, we defined five and three geographic groups for all subsequent analyses, corresponding to *P. grosskopfii* and *P. yuma*, respectively.

To quantify and evaluate the genetic structure and the degree of connectivity of the populations, multiple statistical approaches were used. The first approach was the Bayesian one, which was implemented in STRUCTURE v.2.3.4 software [35] under the admixture model and correlated allel frequencies models. For each possible number of groups (K  =  1–8 for *P. grosskopfii*; K = 1–6 for *P. yuma*), 10 independent runs for 1,000,000 Markov chain Monte Carlo iterations each were performed, discarding 200,000 runs as burn-in. The optimal number of genetic clusters (K) was inferred using the Puechmaille (2016) estimators, which builds upon earlier metrics and provides enhanced visualization tools [36–38]. We applied this method through the StructureSelector web server [39] because it outperforms traditional approaches by more reliably detecting the correct population structure under conditions of uneven or unbalanced sampling. New estimators included the median of medians (MedMed), the maximum medians (MaxMed), the median of means (MedMean), and the maximum of means number of population clusters (MaxMean). Next, we implemented the multivariate statistics of principal component analysis (PCA), using the R package ADE4 [40], ADEGENET [41], and FACTOEXTRA [42]. The PCA is a dimensionality reduction method used to transform and project data points onto fewer orthogonal axes that can explain the greatest amount of variance in the data, allowing for help in population delimitation, without selecting geographic groups a priori to force their grouping or maximize their variances. Likewise, genetic differentiation among geographic sections, and genetic groups suggested by STRUCTURE, was calculated using the standardized statistics Fixation Index (*F*_ST_) [43] using ARLEQUIN v.3.5.2 [44]. Both PCA and *F*_ST_ were performed with neutral, outlier and all SNPs, to identify whether there was any differentiation in the grouping or contribution and strength of presumable outliers/adaptive loci on the structure patterns.

We defined putative genetic stocks for downstream analyses by integrating three lines of evidence, including co-ancestry inference with STRUCTURE, as well as genealogical relationships and historical demography, as described below.

### Phylogeographic relationships

Additionally, a phylogeographical reconstruction analysis was conducted using the neutral, outliers, and combined datasets to provide genealogical support (evidence of reciprocal monophyly) for all the allele frequency-based results already inferred and to evaluate the historical (neutral) and recent (non-neutral) contributions to the observed patterns of genetic structure from a phylogenetic perspective [22]. For this purpose, a maximum likelihood (ML) tree was inferred in RAxML v.8.2.X [45] using the GTR + G evolutionary model, using each type of dataset (neutral, non-neutral, and all loci). For the full ML tree searches, 1000 replicates of the rapid bootstrap algorithm of RAxML were used to account for uncertainty in the estimation of the tree topology [46] in the CIPRES Science Gateway [47]. Phylogenetic trees were independently inferred for each dataset (e.g., neutral and outlier SNPs) or combining them. In RaxML trees the branch lengths represent the number of substitutions per site. Because each dataset differs in the number, composition, and distribution of variable sites, branch length scales are not directly comparable among trees.

### Detecting selection and adaptive variation

To evaluate the existence and contribution of adaptive variation to the observed population structure patterns, a selection detection analysis was performed using BAYESCAN v2.1 [29], employing the same running parameters described above, but inferring selection through the False Discovery Rate (FDR) method. The FDR is defined as the expected proportion of false positives among putative outlier markers. For this, it was set a cutoff of 0.001 (0.1%) to classify loci under selection with high confidence. BAYESCAN outputs were visualized as log10(q-values), such that an FDR of 0.001 threshold corresponds to a value of –3 on the plotted scale. Likewise, positive alpha values were then used to distinguish SNPs under directional selection, whereas negative alpha values were used to detect purifying selection [29]. Although FDRs between 1.4% and 5% are commonly admitted as the percentage of false positives within the loci considered under selection in some studies (*e.g.,* Lotterhos and Whitlock 2014) [48], we wanted to be as rigorous as possible in determining that our loci were under selection, using an FDR of 0.1% equivalent to 1/140–1/500 of the probability of occurrence of false positives used previously in other studies.

We also inferred evidence for selective sweep by estimating the Linkage Disequilibrium (LD) [49]; following the methodology of Escobar et al. (2024) [23]. We applied the standardized index of association (rbarD) for the neutral and adaptive loci datasets, using ‘poppr’ software [32]. The analysis was carried out under two hierarchical levels of genetic variation: including both differentiated stocks (all pops) in the analysis and within each stock or subpopulation, to allow, respectively, for testing the LD under a scenario of positive selection (all biological groups) vs. a non-selective scenario (individual biological groups). The principle of the analysis is that a selective sweep signature is a localized pattern of LD levels, characterized by high LD on each side of a beneficial mutation (adaptive loci) and low LD between loci that are located on different sides of the beneficial allele (loci under neutral variation) [49].

### Genetic diversity and demographic inferences of biological groups (stocks)

The diversity parameters were quantified in terms of observed heterozygosity (Ho) and expected heterozygosity (He), nucleotide diversity (Nd) and Percentage of Polymorphic loci (%P) calculated in ARLEQUIN v.3.5.2 [35] with 100,000 permutations, using neutral loci. In addition, the inbreeding coefficient (*F*_IS_) was calculated in the same program, under similar conditions. The diversity parameters calculations were inferred for each genetic stock observed through STRUCTURE and phylogenetic analyses, avoiding the Wahlund effect on our metrics. Geographical structure affects allele frequencies over space and consequently the proportions of different genotypes in the local populations. It leads to the Wahlund effect, which can be defined as the excess of homozygotes or the deficit in heterozygotes observed in a sample of individuals obtained from a structured population, even when the local populations are randomly mating [50]. The contemporary effective population size (*Ne*) was calculated by each genetic stock using NeEstimator v.2 [51], using the linkage disequilibrium method, assuming 0.02 and 95% confidence intervals of the Jackknifing. This method is particularly robust for population structures with migration rates of <0.1 [52] and hence panmictic populations. Because of this, it was not used to calculate effective population size of the geographic populations, but only for biological (stocks) groups.

In addition, according to the observed genetic structure patterns and biological groups formation through STRUCTURE and PCA analyses, we defined genetic populations/stocks, and then, we quantified the gene flow in terms of the effective number of migrants per generation (Nm) between them following the spirit of Sanchez-Bernal et al. (2023) [53] using the program Migrate v3.6.11 [54]. For this purpose, we tested different migrations models between the paired predefined populations (1:full migration (xxxx); 2:unidirectional (x0xx); 3:unidirectional (xx0x); 4:full isolation (x00x)), and then, we selected the best one by comparing their Marginal Likelihood based on the Bezier approximation score, through the Bayes Factor method [LBF = 2 (ln(Prob(D | Model1) – ln(Prob (D | Model2)]. For Theta and M parameters between populations, we used uniform priors. The search strategy used static heating scheme with four chains at different temperatures (1.0, 1.5, 3.0, 1000000.0) and four replicates of one long chain with 50 million generations, discarding a burn-in of 5 million and sampling every 500 generations. To verify the convergence of parameter estimates, we examined the autocorrelation (<0.2), the effective sample size (ESS > 500) and the correct distribution of the probability density plots for estimates of the parameters. To convert the scaled parameter “M” to biological data in terms of number of effective migrants (Nm), we assumed a conservative generation time of 2.5 years used by Martínez et al. (2022) [28] for both species from the Magdalena-Cauca basin. Then, we assumed a substitution rate of 5.0 × 10−9 mutations per site per year, derived from a geometric mean between the conservative nuclear rate of 1.0 × 10−9 mutations per site per year [55] and the nuclear genomic substitution rate of 2.5 × 10−8 mutations per site per year used for other catfish species [56].

We used the program BEAST v.1.8.4 [57] to investigate patterns of changes in effective population size throughout the coalescent time of the *Pimelodus* species, by using Bayesian Skyline Plot approach. All analyses were run for 50 million generations with a burn-in of 10% initial samples, sampling every 10,000 topologies. We used the HKY+GAMMA model of molecular evolution and a lognormal relaxed molecular clock. Priors were adjusted based on preliminary analyses. Final analyses were repeated two times to assure convergence of estimated parameters, and the independent runs were combined. The absolute time scale of the Bayesian skyline plot was calculated using a substitutions rate explained and justified above for the Migrate software.

### Putative identification of flanking region of the outlier SNPs

Then, the complete RAD-seq loci (contig) containing the outlier SNPs were extracted from the “.filtered.fa” archive generated by DISCOSNPRAD software, previously identified by BAYESCAN, and submitted to BLAST to determine their identity putatively. Given that both *Pimelodus* species are non-model species (without a whole sequenced, assembled, or annotated genome), the only expectation was that our loci would match genomes of phylogenetically close species (catfishes) or, failing that, teleosts. It is understood that homologous genes between different species have suffered mutations in their evolutionary trajectory during the divergence, hence more than similarity, the probability of the expected value (E-value < 0.001) was considered as a criterion for their putative nomination. This estimates the expected number of random alignments with a particular score or better that could be found by chance in a given database search. In other words, it represents the likelihood that a specific sequence alignment is attributable to chancer rather than a true biological relationship between the sequences.

Additionally, we performed an enrichment analysis in the PANTHER V19.0 program [58,59] on its online platform. The analyses were focused solely on shedding light on the biological process to which the set of genes could be associated, those that were previously identified using BLAST for the flanking regions of the outlier SNPs. For this purpose, we implemented the Protocol Update for Large-scale genome and gene function analysis with PANTHER Classification System, developed by Mi et al. (2019) [60], using a generic ID list of genes, *Danio rerio* and *Oryzias latipes* as representative teleost models with well-annotated genomes, due to the limited availability of reference organisms; and selecting a functional classification viewed in graphic charts. Likewise, to facilitate the biological interpretation of molecular functions and biological processes identified using the PANTHER Classification System—which integrates curated annotations from the Gene Ontology database—we employed the Gene Set Enrichment Analyst (GSEA), an artificial intelligence–assisted approach using ChatGPT as an auxiliary interpretative tool. This approach was used exclusively to generate structured summaries of gene functions based on enrichment [61] (https://chatgpt.com/g/g-DDiYl6Gbd-gene-set-enrichment-analyst). The AI tool was not used for data generation, statistical analysis, or hypothesis testing, but solely as an aid for organizing and summarizing functionally annotated information previously inferred from PANTHER.

## Results

### Genomic data processing

A total of 35.79 million unique reads were obtained for *P. grosskopfii* and 25.15 million for *P. yuma*, with an average size of 122 bp. Each individual had on average 559,354 reads for *P. grosskopfii* and 449,081 for *P. yuma*. After extracting SNPs and filtering the VCF by coverage, 55,599 and 118,587 SNPs were retained respectively for *P. grosskopfii* and *P. yuma*. The mean depth per SNP in each individual ranged between 12.78 and 6,364.41 for *P. grosskopfii,* and between 12.54 and 6,043.61 for *P. yuma*. Finally, after filtering by STACKS (population module), we retained 2,475 and 7,611 variable loci (representing equal number of SNPs), respectively for each species, with 10% of missing data both. Datasets with other percentages of missing data [*P. grosskopfii*: 0% (0 SNPs) and 5% (471 SNPs); *P. yuma*: 0% (5 SNPs) and 5% (2,374 SNPs)] showed null or fewer abilities for inferring population structure among groups than the 10% missing data SNPs-matrix for both species in terms of percentage of explained variance for the two first principal components (S2 and S3 Figs), and therefore, this last one matrix was selected for the downstream analyses [28]. Six individuals of *P. grosskopfii* and two of *P. yuma* were removed due to having an excessive amount of missing data (>70%), retaining 58 and 55 individuals, respectively, for subsequent analyses. Next, according to the BAYESCAN analysis for outlier detection (S4 Table; S5 Table), we removed 44 and 15 non-neutral SNPs, retaining 2,431 and 7,596 neutral SNPs, respectively, for *P. grosskopfii* and *P. yuma*. After reviewing for LD, according to the analysis generated by using the ia () function, which calculated the IA overall 2,431 and 7,596 neutral loci in the datasets, the rbarD was 0.1984302 for *P. grosskopfii* and 0.1113031 for *P. yuma* (both rbarD  <  0.2). Therefore, no SNPs were removed from the datasets, which were then used for subsequent population structure, demographic and diversity analyses.

### Population genetic structure

**Fig 2:**
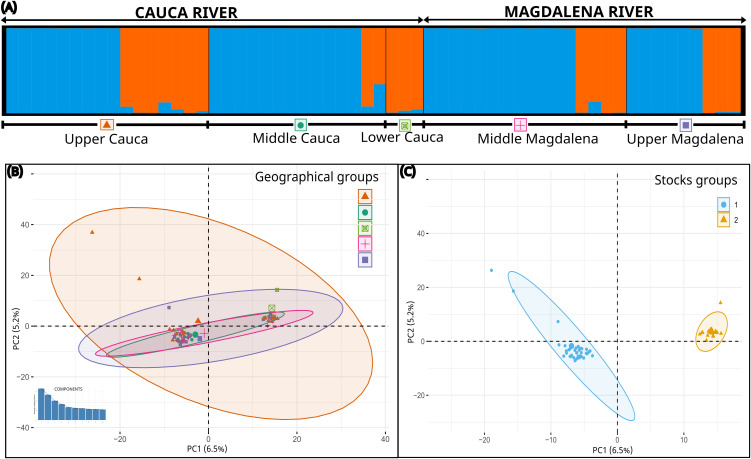
Population structure analysis in *Pimelodus grosskopfii.* **(A)** Coancestry histogram inferred using STRUCTURE for individuals sampled in Upper Cauca, Middle Cauca, Lower Cauca, Middle Magdalena, and Upper Magdalena. **(B)** Principal Component Analysis (PCA) showing the association between geography and population structure. **(C)** PCA showing the association between genetic stocks, as previously identified by STRUCTURE, and the observed population structure. Sample sizes per stock are as follows: Stock 1 (n = 39) and Stock 2 (n = 19).

**Fig 3:**
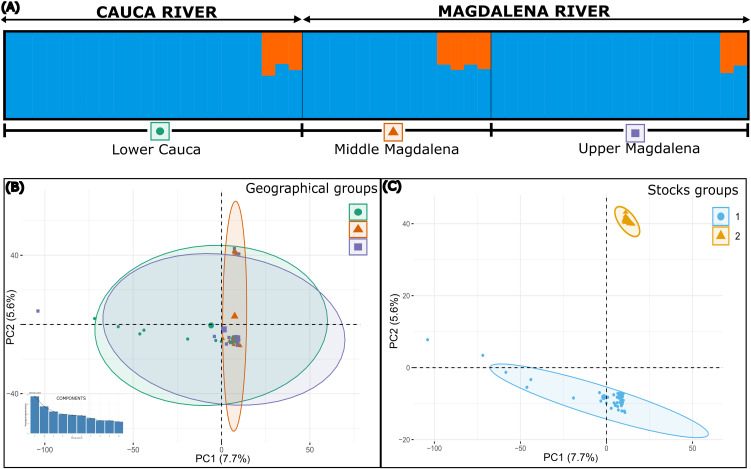
Population structure analysis in *Pimelodus yuma.* **(A)** Coancestry histogram inferred using STRUCTURE for individuals sampled in Upper Cauca, Middle Cauca, Lower Cauca, Middle Magdalena, and Upper Magdalena. **(B)** Principal Component Analysis (PCA) showing the association between geography and population structure. **(C)** PCA showing the association between genetic stocks, as previously identified by STRUCTURE, and the observed population structure. Sample sizes per stock are as follows: Stock 1 (n = 46) and Stock 2 (n = 9).

The structure based on Bayesian analysis revealed two genetic clusters (K = 2) or populations for both species (Figs 2 and 3), which was confirmed by the Puechmaille method (S6 and S7 Figs). These two genetic populations coexist in all sampled sections along the rivers as confirmed by the co-ancestry histogram (Figs 2A and 3A). The structure and genetic differentiation are not shown to be associated with basin geography as confirmed by the PCA analysis (Figs 2B and 3B), but rather with the existence of two differentiated genetic groups (stocks) that, although sharing the same space, showed low genetic interaction (Figs 2C and 3C). This was corroborated by the *F*_ST_ values among geographical areas, which were shown to be non-significant in all cases, ranging between 0.00051–0.02105 for *P. grosskopfii*, and 0.00018–0.00365 for *P. yuma* (P > 0.05) (Table 1). Contrary, the *F*_ST_ between stocks was highly significant for both species, being 0.05677 for *P. grosskopfii*, and 0.10067 for *P. yuma* (P < 0.0001) (Table 1).

**Table 1:** *F*_ST_ values between geographic areas and genetic stocks in the Magdalena-Cauca basin for *Pimelodus grosskopfii* and *Pimelodus yuma,* using neutral (n) and adaptive (o) loci.

Pairwise comparisons	*F*_ST_
*Pimelodus grosskopfii*	
GP1 vs GP2 (n)	0.0060
GP1 vs GP3 (n)	0.0211
GP1 vs GP4 (n)	0.0055
GP1 vs GP5 (n)	0.0005
GP2 vs GP3 (n)	0.0020
GP2 vs GP4 (n)	0.0016
GP2 vs GP5 (n)	0.0009
GP3 vs GP4 (n)	0.0109
GP3 vs GP5 (n)	0.0131
GP4 vs GP5 (n)	0.0030
Stock1 vs Stock2 (n)	0.0568***
Stock1 vs Stock2 (n + o)	0.0696***
Stock1 vs Stock2 (o)	0.5191***
*Pimelodus yuma*	
GP3 vs GP4 (n)	0.0008
GP3 vs GP5 (n)	0.0002
GP4 vs GP5 (n)	0.0037
Stock1 vs Stock2 (n)	0.1007***
Stock1 vs Stock2 (n + o)	0.1049***
Stock1 vs Stock2 (o)	0.7629***

Note: ***Denotes a highly significant probability (P < 0.0001). GP1: Upper Cauca, GP2: Middle Cauca, GP3: Lower Cauca, GP4: Middle Magdalena, GP5: Upper Magdalena.

Finally, given that at least two of the three lines of evidence were concordant in delineating genetic stocks in *P. grosskopfii* and *P. yuma*, we infer that both species comprise two distinct stocks. This inference is supported by the evidence of historical divergence in evolutionary trajectories (neutral genealogies), reciprocal monophyly (neutral+outlier genealogies), distinct demographic histories (see below), and differentiated co-ancestry patterns, the latter being particularly defined only in *P. grosskopfii*.

### Genealogical relationships

**Fig 4:**
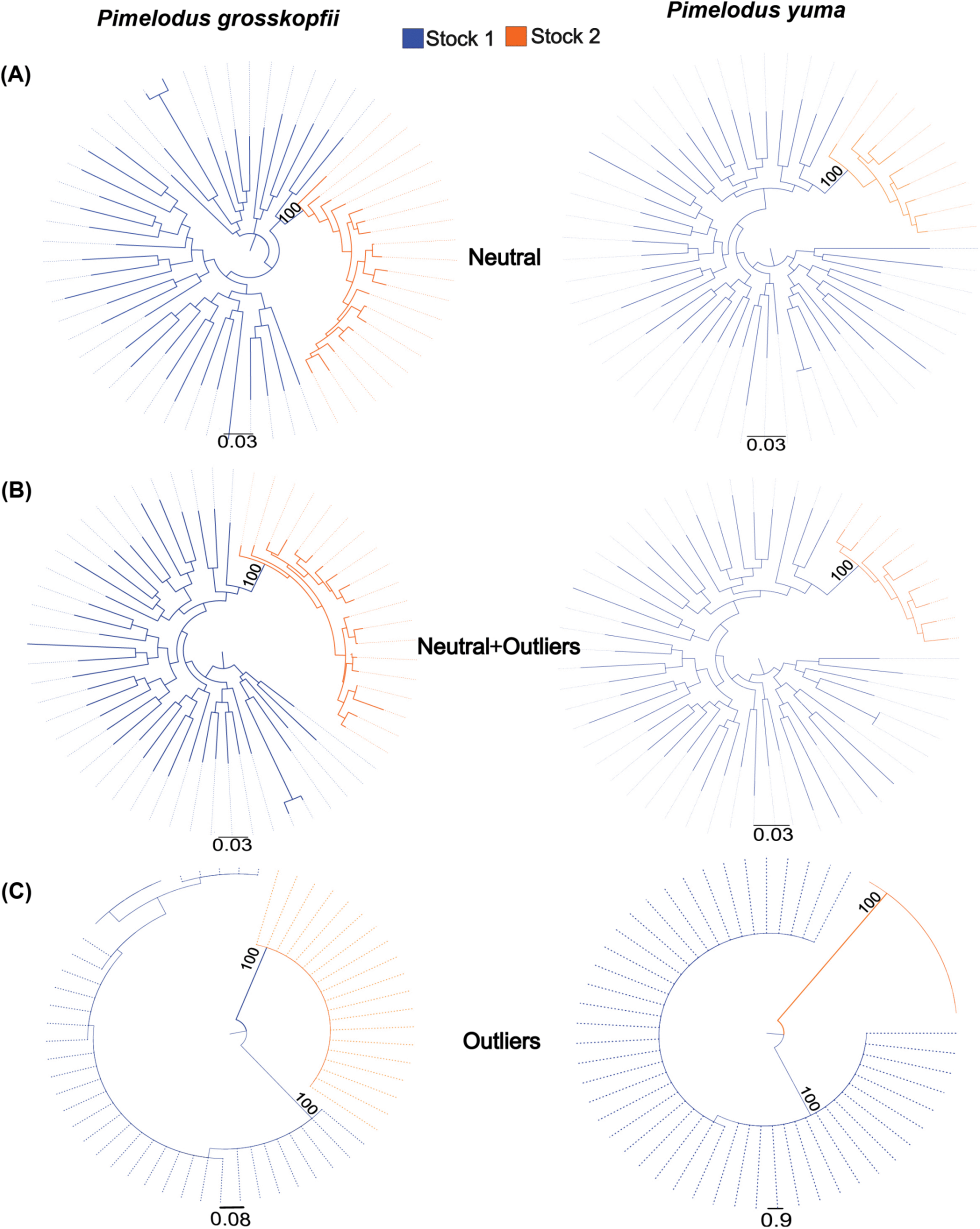
Phylogeographic reconstruction analysis for *Pimelodus grosskopfii* and *P. yuma* in the Magdalena–Cauca basin, based on Maximum Likelihood inference using RAxML. Analyses were performed using (A) neutral loci, (B) neutral + outliers loci, and (C) outlier loci only. Blue indicates Stock 1, and orange indicates Stock 2.

The phylogeographical approach based on the genealogical analyses performed with each dataset revealed different relationship patterns between stocks (Fig 4). When using the “neutral” or “neutral+outliers” datasets, it was consistently demonstrated that the putative stocks are not statistically reciprocal monophyletic groups (Fig 4A and 4B). However, a reciprocal monophyly pattern was observed between stocks when only the outlier loci were used, showing two well-supported groups (100%), each one represented by a genetic stock (Fig 4C). For both species, in the phylogenetic reconstruction involving neutral SNPs, the putative stock 2 was shown as a subpopulation derived from the stock 1, which was, in turn, the ancestral one or basal population.

### Selection and adaptive variation

**Fig 5:**
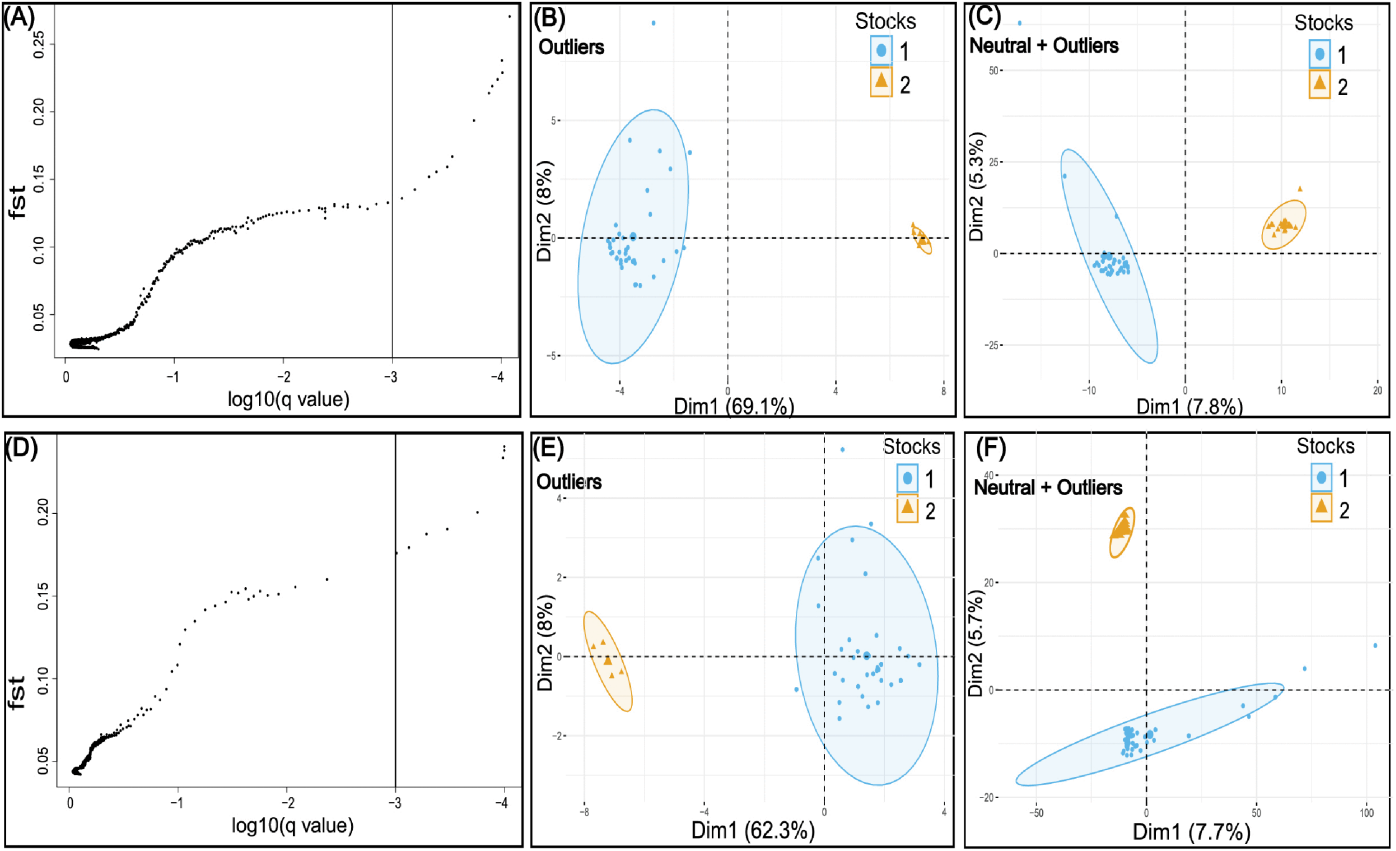
Natural selection and adaptive variation analysis in *Pimelodus grosskopfii* and *P. yuma* from the Magdalena–Cauca basin. (A) and (D) show BAYESCAN analyses of 2,475 and 7,611 single nucleotide polymorphisms (SNPs) for *P. grosskopfii* and *P. yuma*, respectively. The vertical axis represents locus-specific *F*_ST_ values, while the horizontal axis shows the logarithm of Q-values. The vertical black line indicates the threshold used for outlier detection (false discovery rate (FDR) = 0.001), represented as log10(q-value) = –3. Each dot corresponds to an individual SNP. Outlier SNPs under positive selection are located above the upper-right threshold line. (B) and (E) illustrate Principal Component Analyses (PCA) based on outlier loci for *P. grosskopfii* and *P. yuma*, respectively. (C) and (F) show PCA results using both neutral and outlier loci for *P. grosskopfii* and *P. yuma*, respectively.

According to BAYESCAN, the selection was detected as an evolutionary force acting on the populations, at the very conservative value for FDR of 0.001 (0.1%) cut-off (Fig 5A and 5D), providing strong evidence consistent with its existence and strength. In addition, the type of selection signature was consistent with directional, according to the positive alpha values (S4 and S5 Tables) (negative values are associated with purifying selection). Likewise, the PCA, using “outlier” loci, showed a similar pattern of two well-defined stocks, with a percentage of explanation of the total variance for the first two components of 77.1% for *P. grosskopfii*, and 70.3% for *P. yuma*. However, when “neutral+outlier” loci datasets were included for the same analysis, the percentage of explanation of the variance decreased drastically to 13.1% and 13.4%, respectively for each species (Fig 5B-5C, Fig 5E-5F). A similar pattern was observed in the *F*_ST_ estimated between stocks using outliers loci, which showed a very strong and highly significant (P < 0.0001) fixation index of 0.51914 for *P.* *grosskopfii*, and 0.76289 for *P. yuma,* decreasing drastically to non-significant (P > 0.05) values of 0.06958 and 0.10486, respectively for each species (Table 1) when “neutral+outlier” loci datasets were included for the analysis.

As a selective sweep signature, the LD, inferred through the standardized index of association (rbarD), showed, as expected under a genomic selective sweep scenario, a high LD for both *P. grosskopfii* (rbarD = 0.6781) and for *P. yuma* (rbarD = 0.6050) on each side of a beneficial mutation (adaptive/outliers loci); and a low LD for both [*P. grosskopfii* (rbarD = 0.0212); *P. yuma* (rbarD = 0.0746)] between loci that are located on different sides of the beneficial allele (loci under neutral variation) (Table 2). However, when each population was evaluated separately [removing the effect of differential environments or adaptive factors (directional selection bias)], the LD was very low in both the stock 1 [rbarD (neutral) = 0.0158–0.0546; rbarD (adaptive) = 0.1256–0.1651] and stock 2 [rbarD (neutral) = 0.0032–0.0243; rbarD (adaptive) = 0.0746–0.2750], for *P. grosskopfii* and *P. yuma* (Table 2), suggesting that the signals of selective sweep are explained by a force acting differentially between stocks.

**Table 2:** Linkage disequilibrium (LD) inferred through the standardized index of association (rbarD) for neutral and outlier (putative adaptive) loci datasets, under two hierarchical levels of genetic variation: including both differentiated genetic stocks in the analysis and within each stock or subpopulation.

	*Pimelodus grosskopfii*	*Pimelodus yuma*
	rbarD	rbarD
**Stock 1 + Stock 2**		
Neutral loci	0.0212	0.0746
Outlier loci	0.6781	0.6050
**Stock 1**		
Neutral loci	0.0158	0.0546
Outlier loci	0.1651	0.1256
**Stock 2**		
Neutral loci	0.0243	0.0032
Outlier loci	0.0746	0.2750

Note: a selective sweep signature is a localized pattern of linkage disequilibrium (LD) levels [49], characterized by high LD (*e.g.* rbarD > 0.5) on each side of a beneficial mutation (adaptive/outlier loci) and low LD (*e.g.* rbarD < 0.2) between loci that are located on different sides of the beneficial allele (loci under neutral variation) [49].

### Genetic diversity and demographic inferences

The genetic diversity also revealed differences between the two stocks in the *Pimelodus* species. The observed heterozygosity for both species was always slightly lower in Stock 1 (0.28–0.31) than in stock 2 (0.44–0.52) (Table 3); being contrary to the behavior of expected heterozygosity, which was slightly similar for all stocks throughout the species (0.31–0.37) (Table 3). In relation to the inbreeding index (*F*_IS_) for both species, this was between 8.7% − 9% in stock 1, while it showed an excess of heterozygotes, with negative values, for Stock 2 (Table 3). Regarding the effective population size (*Ne*), it could only be estimated in stock 1 of both species, due to the sensitivity of the algorithm with small sizes of the sample in which it is estimated, as the case of stock 2. Thus, in *P. grosskopfii* the *Ne* reached 379.3, while in *P. yuma* it was 313.8.

**Table 3:** Diversity indexes and demographic parameters for genetic stocks of *Pimelodus grosskopfii* and *Pimelodus yuma* in the Magdalena-Cauca basin.

	*Pimelodus grosskopfii*		*Pimelodus yuma*	
	STOCK 1_(n = 39)_	STOCK 2_(n = 19)_	STOCK 1_(n = 46)_	STOCK 2_(n = 9)_
**Ho**	0.28498	0.43901	0.30807	0.52369
**He**	0.31119	0.31895	0.32080	0.37468
***F***_**IS**_	0.09010	−0.37210	0.08720	−0.42180
**Nd**	0.29140	0.31106	0.275301	0.335344
**%P**	95.97	97.53	90.89	89.11
***Ne***	379.3 (134.3-∞)	∞(∞-∞)	313.8 (92.8-2702.5)	∞(∞-∞)
**2Nm (Stock1 → Stock2)**	0		2.74 (0.600–4.900)	
**2Nm (Stock2 → Stock1)**	4.30 (2.030 - 6.700)		5.99 (3.386–8.650)	

Note: Ho = Observed Heterozygosity, He = Expected Heterozygosity, *F*_IS_ = Endogamic index, Nd = nucleotide diversity, %P = Percentage of Polymorphic loci, *Ne* = Effective population size, 2Nm = number of migrants per generation (*Pimelodus* generation time is ~ 2.5 years).

After the migration model tests between stocks in Migrate-n, we found that in *P. grosskopfii*, the unidirectional pattern was the best model explaining migration between stocks (lmL = −36156,82), in this case, from stock 2 toward stock 1 (2Nm = 4.3 migrants per generation, S8 Table; Table 3); while the bidirectional model was the best one explaining migration between stocks in *P. yuma* (lmL = −23576,68), with 2.74 migrants per generation from the stock 1 toward stock 2, and 5.99 migrants per generation from stock 2 toward stock 1 (S8 Table; Table 3).

**Fig 6:**
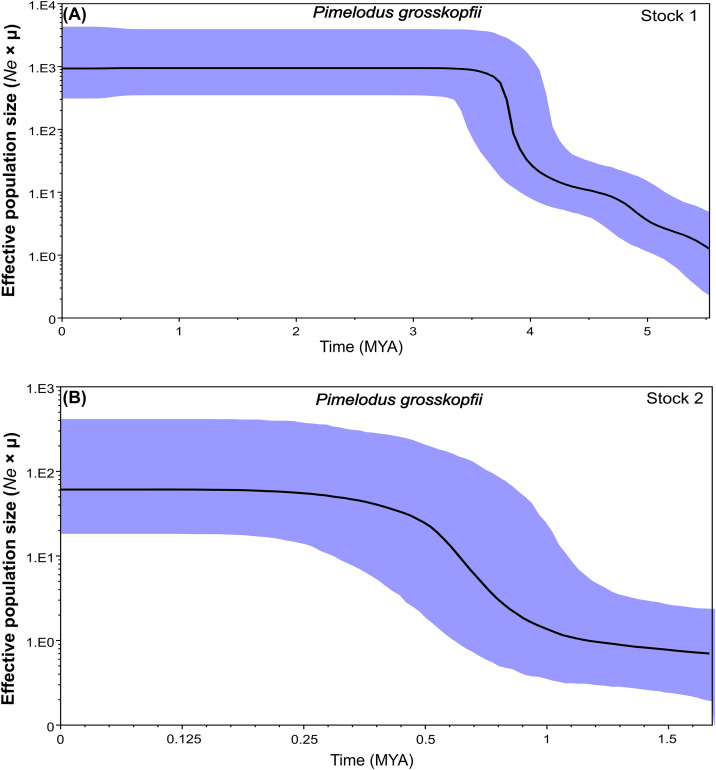
Bayesian Skyline Plot (BSP) for *Pimelodus grosskopfii* populations from the Magdalena–Cauca basin. **(A)** BSP for Stock 1. **(B)** BSP for Stock 2. The Y-axis represents the effective population size scaled by the mutation rate (*Ne* × μ), while the X-axis represents time in millions of years before present (MYA). The shaded area represents the 95% highest posterior density interval.

**Fig 7:**
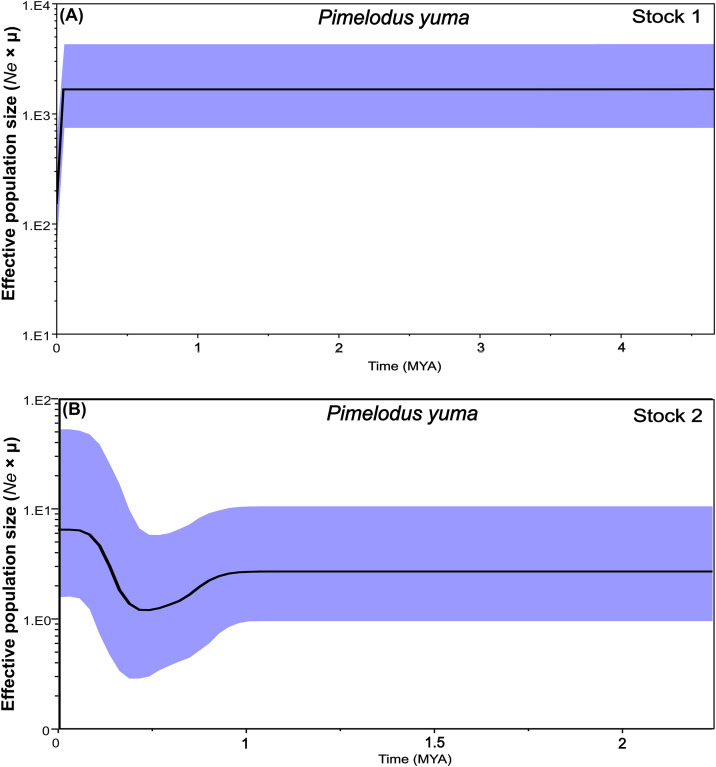
Bayesian Skyline Plot (BSP) for *Pimelodus yuma* populations from the Magdalena–Cauca basin. **(A)** BSP for Stock 1. **(B)** BSP for Stock 2. The Y-axis represents the effective population size scaled by the mutation rate (*Ne* × μ), while the X-axis represents time in millions of years before present (MYA). The shaded area represents the 95% highest posterior density interval.

Likewise, the Bayesian skyline plot (BSP) (Figs 6 and 7) showed that the most recent common ancestor for stock 1 in both species was detected between 4.5–5.5 MYA (Late Miocene-Pliocene), suggesting an older origin for this stock. From there, in *P. grosskopfii*, stock 1 experienced a population expansion until ~3.5 MYA, when it stabilizes and remains constant until the present (Fig 6A). In the case of *P. yuma*, stock 1 did not experience expansion but remained constant until ~0.125 MYA (Fig 7A), from which time it suffers a reduction in population size until the present, which, even reduced, remained higher than that of stock 2. Particularly, in the case of stock 2 for both species, their most recent common ancestor was detected between ~1.7–2.5 MYA (Early Pleistocene). From ~1.2 MYA (Middle Pleistocene), in *P. grosskopfii*, stock 2 experienced a population expansion until ~0.24 MYA (Late Pleistocene), when it already stabilizes and remains constant until the present. In the case of *P. yuma*, stock 2 does not experienced expansion but remains constant from its detection until ~1.0 MYA (Middle Pleistocene), a time from which, unlike *P. grosskopfii*, it suffers an abrupt reduction in population size until 0.4 MYA, with a subsequent rapid expansion that goes up to ~0.24 MYA (Late Pleistocene), from which time it stabilizes until the present.

### Putative identity of selective SNPs loci and enrichment analyses

After performing identity analyzing in BLAST (GenBank), only six of the 44 SNPs for *P. grosskopfii* [11338, 51741, 52924, 54464, 54596, 54676 (see S9 Table)] could be attributed to a genic or proteinic identity [E3 ubiquitin/ISG15 ligase TRIM25 (TRIM25), E3 ubiquitin-protein ligase (HERC2), ATPase Phospholipid Transporting 10D (ATP10D), RAS p21 protein activator 4 (RASA4), Neurexin 1 (NRXN1), and, GTPase activating protein (SH3 domain) binding protein 1 (G3BP1)], with 86–100% of identity (E-value<0.00001). In the case of *P. yuma*, of the 15 SNPs tested, only two [8894, 71235 (see S10 Table)] found a match with a genic region [SH3 domain-containing kinase-binding protein 1 (SH3KBP1), Ribosomal Oxygenase 2 (RIOX2)], with 92–93% of identity (E-value<0.00001). In addition, the PANTHER results showed three molecular functions where the *P. grosskopfii* putative genes could be involved in (ATP-dependent Activity, Binding and Transporter activity) (see S11A Fig), and four biological processes (Biological regulation, cellular process, localization, and metabolic process) (see S11B Fig). In the case of *P. yuma*, only one molecular function could be detected (Catalytic activity) (see S11C Fig), as well as only one biological process (Cellular process) (see S11D Fig).

Finally, functional annotations derived from the PANTHER Classification System, corroborated with classifications from the Gene Ontology (GO) database and supported by AI-assisted interpretation (GSEA) indicated that the identified genes are associated with several well-characterized physiological processes. Specifically, TRIM25-like proteins are involved in ubiquitination and antiviral immune responses; RASA4 participates in Ras-mediated signaling pathways; G3BP1 is associated with stress granule formation during cellular stress; NRXN1 is involved in synaptic communication and neuronal signaling; ATP10D plays a role in phospholipid transport and membrane dynamics; and HERC2 is associated with ubiquitination, DNA repair, and cellular stress responses.

In *P. yuma*, SH3KBP1 is involved in intracellular signaling and vesicular trafficking, while RIOX2 participates in epigenetic regulation through histone modification and cellular differentiation processes.

## Discussion

This study investigated the genetic structure of *Pimelodus grosskopfii* and *P. yuma* within the Magdalena–Cauca Basin through a genomic approach integrating both coalescent-based and allele frequency-based methods. The results revealed no evidence of spatial genetic structuring. Instead, individuals from both species were assigned to two nearly discrete genetic stocks that maintain a degree of gene flow and coexist throughout the basin, occupying the same riverine habitats. This confirms the findings of Joya et al. (2021) [7] and Restrepo-Escobar et al. (2021) [8]. These findings demonstrate that the large-scale geographic structure is not the main driver of genetic diversity distribution across the basin and are consistent with previous observations in the Cauca River [7,8]. Selection emerged as the most plausible historical force shaping these stocks. The observed genetic structuring could be explained by isolation driven by differential adaptation to environmental cues influencing reproductive migration, a mechanism known to promote population divergence and reproductive isolation [16]. This supports the rejection of neutral variation as the sole driver of the observed genetic patterns. Based on phylogenetic, demographic, and genetic diversity evidence, each stock should be considered a distinct Management and Adaptive Unit [22], with stock 2 being the derived and more recent population—potentially the most vulnerable to future environmental or anthropogenic pressures within the basin.

### Population genetic structure and the role of selection and neutral variation

Our results indicate high genetic connectivity for *Pimelodus yuma* and *P. grosskopfii* populations, suggesting that physical barriers do not significantly restrict gene flow across their examined distribution. Thus, a simple model of genetic differentiation structured by geographic distance (isolation-by-distance) is rejected, despite the broad sampling range of more than 1,600 km for *P. grosskopfii* and 1,200 km for *P. yuma*. Even at this broader scale, our analysis corroborates previous findings [7,8], confirming the coexistence of two genetic stocks in sympatry. This two-stock structure persists despite the high gene flow observed in each stock across the sampled geographic regions, suggesting that differentiation is driven by non-spatial factors, such as ecological adaptation.

Our findings suggest that these stocks likely originated from ecological adaptations, with Stock 2 representing a strongly selected subset derived from a larger, more diverse ancestral population (Stock 1). This scenario aligns with hypotheses of divergence via “migratory timing” [16], where gene flow occurs only among individuals successfully reproducing after reaching spawning grounds, modulated by environmental cues [55]. The concept of temporal isolation as a driver of differentiation is not novel for this genus; it had been previously suggested for both *P. yuma* [7] and *P. grosskopfii* [8], linked to the basin’s distinct seasonal and hydrological periods. This hypothesis is supported by key biological observations. For instance, *Pimelodus* exhibits peaks of ichthyoplankton presence only during rainy seasons in the Magdalena River basin [15]. Furthermore, a greater number of *P. grosskopfii* individuals have been observed participating in the migration during the first of the two main hydrological periods in the Magdalena River [3]. However, conclusive genetic evidence for such temporal structuring remains to be demonstrated.

Evidence from allopatric and sympatric breeding populations shows that differences in migratory behavior often coincide with genetic divergence [16]. Mechanisms in several animals like selection against intermediate phenotypes can reinforce reproductive isolation [16]. In the case of *Pimelodus,* we hypothesize that extreme phenotypes—ecotypes with migration and spawning timing optimized for one flood pulse—achieve high fitness via synchronized reproduction and resource exploitation. However, individuals with intermediate traits probably suffer timing mismatches, reduced offspring survival, and a fitness valley, reinforcing divergence and reproductive isolation amid gene flow. Though not fully understood, behavioral divergence in migration likely plays a role in population differentiation. This has been demonstrated in Pimelodidae species, where migration and reproductive cycles influence gene flow, as observed in *P. maculatus* across Uruguay, Tibagi, and Tietê rivers [62–64], and in *Pseudoplatystoma corruscans* in the São Francisco and Paraguay basins [64].

Interestingly, despite no spatial structure, nearly discrete genetic stocks occupying the same river sections during different or overlapping migratory periods have been reported in *P. reticulatum* in the Paraná–Paraguay Basin, and in *P. grosskopfii* and *P. yuma* in the Cauca River [7,8]. These cases highlight the role of seasonal migration in population divergence and reproductive isolation [16,17]. Studies have linked such migratory divergence to speciation, often resulting in strong genetic differentiation—as seen in sharks, sea turtles, salmonids, and warblers [16].

A recent study on *Cyphocharax magdalenae*, a Characiform species co-occurring with *Pimelodus*, also revealed a seasonal population structure comprising two genetic stocks [20]. In that study, sampling was seasonally stratified, supporting the association of one stock with dry seasons (Jan–Mar, Jul–Aug) and the other with rainy seasons (Apr–Jun, Oct–Dec). These findings provide additional evidence for the presence of temporal genetic structuring in Neotropical rivers, further supporting the patterns of seasonal divergence observed in our current assessment.

Although additional seasonally targeted sampling is needed for *P. grosskopfii* and *P. yuma* to confirm seasonal stock association (e.g., rainy–dry or rainy–rainy cycles), our study clearly shows a selective contribution to stock divergence. Outlier loci showed fixed alternative alleles between stocks (≥90% in one and ≤10% in the other), with *F*_ST_ and PCA variance increasing nearly sevenfold when adaptive loci were included. Moreover, linkage disequilibrium rose when both neutral and adaptive markers were analyzed jointly. In addition, removing adaptive loci from the SNP matrix slightly reduced structure, and admixture was observed in some individuals, yet overall differentiation remained.

Across multiple fish systems, selection has emerged as a recurrent mechanism promoting reproductive isolation and even speciation, operating through adaptive divergence in ecological traits, life-history strategies, and mating-related phenotypes [65–67].

Phylogenetic analysis using neutral loci confirmed this structure as historical. Stock 2 formed a well-supported distinct monophyletic group, suggesting long-term fixation of alleles and an independent evolutionary trajectory. Interestingly, phylogenetic evidence suggested that Stock 2 likely emerged from a dispersal event originating in Stock 1 rather than from vicariance, possibly accelerated by past climatic shifts. According to Block & Levine (2021) [68], climate-driven environmental changes can promote the dispersion and establishment of genotypes better suited to emerging local conditions.

### Chronology of the selection and the genetic structuring

It is evident that a selective force plays a central role in shaping the genetic structure of both *Pimelodus yuma* and *P. grosskopfii*. Available evidence indicates that this selective pressure has likely operated persistently over evolutionary timescales, progressively intensifying in concert with the evolving climatic and hydrological configuration of the Magdalena basin, resulting in increasingly restricted gene flow between individuals from the two stocks up to the present. This pattern is supported by multiple lines of neutral genetic variation analyses, including principal component analysis (PCA), pairwise *F*_ST_ estimates, migration rate inferences from MIGRATE, ancestry proportions from STRUCTURE (albeit partially), and historical phylogeographic reconstruction using RAxML. A key indicator of historical divergence is the time of origin of Stock 2, which appears to have emerged more recently than Stock 1, as confirmed by phylogenetic reconstruction. The most recent common ancestor (MRCA) of Stock 2 in both species is estimated to date back to ~2 million years ago (Middle Pleistocene), indicating a substantial evolutionary trajectory despite its younger age compared to Stock 1 (4.5–5.5 MYA).

Additionally, Bayesian Skyline Plot reconstructions revealed a recent demographic expansion in Stock 2 for both species, estimated between 0.25–0.5 MYA for *P. yuma*, and 0.25–1 MYA for *P. grosskopfii*. This pattern suggests a recent intensification of selective pressure—likely linked to evolving climatic and hydrological conditions in the Magdalena basin—favoring adaptive genotypes with enhanced resource exploitation and environmental performance [69]. Once adaptation occurs, fitness advantages may arise from changes in life-history traits that enhance population growth [69]. Functionally, these advantages may stem from increased or more efficient resource utilization, with direct consequences for demographic trends.

Interestingly, the timing of these expansions aligns with major climatic fluctuations during the Pleistocene [70], particularly those associated with abrupt reorganizations of atmospheric circulation driven by latitudinal shifts of the Intertropical Convergence Zone (ITCZ) [71]. During glacial periods, the ITCZ shifted equatorward, while in interglacial it moved northward in response to Northern Hemisphere warming [72].

Although the ITCZ is a global phenomenon, its effects on precipitation and drought patterns are modulated by regional atmospheric and ecological conditions. Equatorial rainfall is not governed solely by ITCZ convergence, but also by local factors such as topography-induced convection, proximity to oceans, atmospheric jets and waves, moisture recycling, and variability in land cover and albedo [73,74]. In South America, the Andean uplift and atmospheric CO₂ decline significantly influenced the positioning of the ITCZ, as well as the evolution of the South American monsoon system and rainforests [75].

Indeed, the estimated formation periods of Stock 1 (~4–5 MYA) and Stock 2 (~1.8–2.5 MYA) coincide with two major episodes in the final geomorphological reconfiguration of the Magdalena Basin, driven by the last phases of the Andean orogeny. The first event involved the separation of the Magdalena Basin from the Maracaibo Basin [≤4.5 MYA; [76]], and the second from the Orinoco Basin [~2.5 MYA; [77]], contributing to the long-term geographic and hydrological isolation of its ichthyofauna, and likely also to a new regional climate reorganization.

Currently, the Magdalena–Cauca basin exhibits remarkable morphological, ecological, and climatic heterogeneity, shaped by extensive floodplains, high Andean ranges, and localized climatic events, which result from interactions between orographic features and seasonal rainfall influenced by the Pacific Ocean and moisture inflows from the Amazon and Orinoco basins [78]. Critically, the biannual passage of the Intertropical Convergence Zone (ITCZ) generates two rainy seasons per year [78], leading to major flooding periods that drive dual cycles of biological productivity in floodplains through increased water and sediment input [79]. This seasonal pattern triggers two annual peaks of reproductive migration in potamodromous fishes such as *Pimelodus* [15].

### Rainy seasons and the probably asynchronic migratory response modeling stocks in *Pimelodus*

We hypothesize that the two rainy seasons in the Magdalena–Cauca Basin are key drivers of the observed selection patterns, potentially promoting reproductive isolation via asynchronous migratory behavior in Stock 2 (respect to the Stock 1) of both *Pimelodus* species. While a deeper genomic investigation is needed to confirm this, several lines of evidence support it: (i) ichthyoplankton from both species peaks during the rainy seasons [15], indicating biannual reproduction; (ii) captive observations suggest different individuals spawn in each peak (Atencio-García, 2025, pers. comm.); and (iii) evidence of putative selection on SNPs located near genes potentially associated with physiological processes underlying reproductive migration, including environmental stress, neuro-sensory pathways, immune modulation, and metabolic regulation, as identified in this study and previously reported in other migratory fishes [80–82].

The two largest cis-Andean South American river basins harboring the greatest diversity of *Pimelodus* species—the Orinoco and Amazon—exhibit a unimodal flood-pulse regime associated with reproductive migration in *Pimelodus* species [83–85]. In contrast, the Magdalena–Cauca basin exhibits bimodal regime due to ITCZ dynamics, with a second rainy season and flood-pulse (Oct–Dec) aligning with South American cis-Andean basins, and a first rainy season (Apr–Jun) unique to this trans-Andean River system [3,14,15]. Given the cis-Andean origin of *P. grosskopfii* and *P. yuma* ancestors [28], adaptation to respond reproductively to this first flood peak may be a recent, trans-Andean region-specific event. The emergence of Stock 2 likely reflects an evolutionary response to this novel environmental cue, as suggested by phylogenetic and demographic reconstructions showing a younger, dispersed derived lineage from Stock 1—the more diverse and ancestral population. It is known that dispersal evolution and local adaptation are eventually linked, affecting the range dynamics of species facing environmental changes associated with climate [68]. Genetic diversity and demographic analyses reveal lower heterozygosity and effective population size in Stock 2, consistent with strong selection effects [86,87]. Similar signatures of selection-driven divergence have been reported in *Oncorhynchus nerka* and *Salmo trutta* [88].

We hypothesize that this kind of striking selective variation in migration patterns probably modelling structure in *Pimelodus,* is a well-known mechanism of ecotype differentiation called “migration timing” [89–92] and could have a genetic base behind. For instance, in species like *Chinook salmon*, early and late migrants show distinct phenotypes (ecotypes) linked to regions near genes like *ROCK1* and *GREB1L* [17]. While in salmonids a few genes explain most of the variation (85%) [88], in *Pimelodus*, adaptive divergence appears polygenic, with loci under selection accounting for ~70% of the variance, through an apparent selective sweep mechanism, as suggested by the LD test (rbarD) by using neutral and selective loci.

Interestingly, in this study, although gene flow exists, population structure remains. In our dataset, only ~15% of selective loci showed fixation patterns expected under Isolation by Adaptation (IBA) [65], while 67% showed high heterozygosity in one stock and homozygosity in the other—suggesting balancing selection or ongoing gene flow (See S12 and S13 Figs for more details). This aligns with the “isolation with migration” model [93,94], where likely the selection and drift act within populations, but gene flow counteracts divergence. A similar pattern was observed in Chinook salmon, where lineages or populations appear to be more genetically distinct than expected, despite clear evidence of interbreeding among them [92].

STRUCTURE analyses reveal that *P. grosskopfii*’s Stock 2 is highly differentiated (with <15% shared ancestry with Stock 1), likely due to restricted, unidirectional migration, while *P. yuma*’s Stock 2 (~50% shared ancestry) reflects more bidirectional gene flow. This differentiation may reflect either stronger selection in *P. grosskopfii* or a longer divergence time (~1.7 MYA) compared to *P. yuma* (~1 MYA), consistent with greater neutral allele drift over time [65] in *P. grosskopfii*. In addition, this pattern may also be explained by the retention of ancestral polymorphisms, whereby shared genetic variation inherited from a common ancestor can persist across populations after divergence, generating signals of coancestry even in the absence of ongoing gene flow [86,93]. As despite observable coancestry in *P. yuma* between the putative stocks, phylogenomic inferences support a historical differentiation between them.

Lastly, sampling year was excluded as a confounding structuring factor, given that individuals from both genetic stocks were often collected concurrently in the same sampling year, as shown in S1 Table.

### Implications for conservation

Based on our findings, genetic Stocks 1 and 2 within each species should be regarded as both Management Units (MUs) and Adaptive Units (AUs), considering the consistent phylogenetic patterns and population structure derived from both neutral and adaptive loci [22,95]. Each stock represents a distinct lineage with ongoing gene flow or demographic connectivity.

Stock 1, in both species, shows higher abundance, older evolutionary persistence, and more stable diversity indices, as well as moderate effective population size (*Ne* = 50–500) [96], indicating a reduced risk of severe inbreeding effects in the short term. It is thus the more resilient and genetically healthy stock. In contrast, Stock 2 appears younger, less frequent in the basin, and under stronger selective pressure. Although its *Ne* could not be directly estimated, Bayesian Skyline Plots suggest a smaller effective size relative to Stock 1. These features suggest Stock 2 is at higher risk of inbreeding depression and reduced reproductive viability (*Ne* < 50–100), making it a conservation priority [96–98].

Nonetheless, neither stock meets the minimum *Ne* threshold (>500) required to ensure long-term adaptive potential [96,98], meaning both are below the level needed to maintain evolutionary resilience. These observations warrant caution, and further studies with increased sample sizes are recommended to confirm the current patterns.

Additionally, diversity indices were lower compared to previous studies using microsatellites in the same basin for both species [7,8]. For instances, in *P. grosskopfii*, observed heterozygosity was 43.4% lower and the inbreeding coefficient was 44.8% lower than estimates obtained using microsatellites. Similarly, *P. yuma* showed reductions of 42.5% in heterozygosity and 41.0% in the inbreeding coefficient relative to microsatellite-based estimates. These differences reflect the biallelic nature of SNPs, which typically underestimate genetic diversity compared to multiallelic microsatellites [99]. Therefore, a correction factor of approximately 41–45% may be considered when comparing diversity estimates between marker types, particularly when interpreting SNP-based results regarding these species. Nevertheless, SNPs offer higher genomic resolution and reduced bias in estimating population parameters by capturing both neutral and adaptive processes, making them a powerful complement to SSRs in conservation genetics [95].

## Conclusion

Our findings corroborate that *Pimelodus grosskopfii* and *P. yuma* each harbor two coexisting genetic stocks within the Magdalena–Cauca Basin, uncorrelated to geography, and shaped by both neutral processes and, more importantly, adaptive divergence. This divergence, likely driven by the basin’s bimodal hydrological regime and historical environmental pressures, supports the recognition of each stock as both a Management and Adaptive Unit. These results underscore the importance of incorporating evolutionary and ecological processes into conservation planning to safeguard the long-term persistence of these endemic migratory species amid ongoing anthropogenic threats.

## Supporting information

S1 Table Sampling sites, origin, and collection details of *Pimelodus grosskopfii* and *P. yuma.* (XLSX)

S2 Fig Percentage of variance explained by principal components in the SNP-based PCA of *Pimelodus grosskopfii.* (PDF)

S3 Fig Percentage of variance explained by principal components in the SNP-based PCA of *Pimelodus yuma.* (PDF)

S4 Table Bayescan analysis for *Pimelodus grosskopfii.* (TXT)

S5 Table Bayescan analysis for *Pimelodus yuma.* (TXT)

S6 Fig Puechmaille analysis for *Pimelodus grosskopfii.* (PDF)

S7 Fig Puechmaille analysis for *Pimelodus yuma.* (PDF)

S8 Table Bayes factor–based support for alternative migration models inferred in MIGRATE for *Pimelodus grosskopfii* and *Pimelodus yuma.* (XLSX)

S9 Table Outlier SNPs identified by stock and nucleotide sequences of their flanking regions for *Pimelodus grosskopfii.* (XLSX)

S10 Table Outlier SNPs identified by stock and nucleotide sequences of their flanking regions for *Pimelodus yuma.* (XLSX)

S11 Fig Gene Ontology (GO) enrichment analysis inferred using PANTHER in *Pimelodus grosskopfii* and *Pimelodus yuma.* (PDF)

S12 Fig Genotypic frequencies of adaptive loci across stocks in *Pimelodus grosskopfii.* (PDF)

S13 Fig Genotypic frequencies of adaptive loci across stocks in *Pimelodus yuma.* (PDF)
