# Supplementary figures and images for: Genomic insights into population structure and adaptive variation of Pimelodus yuma and Pimelodus grosskopfii in the Magdalena-Cauca Basin
Source: PLoS One. 2026 Jun 5;21(6):e0351301. doi: 10.1371/journal.pone.0351301 (PMC13240932; doi:10.1371/journal.pone.0351301)

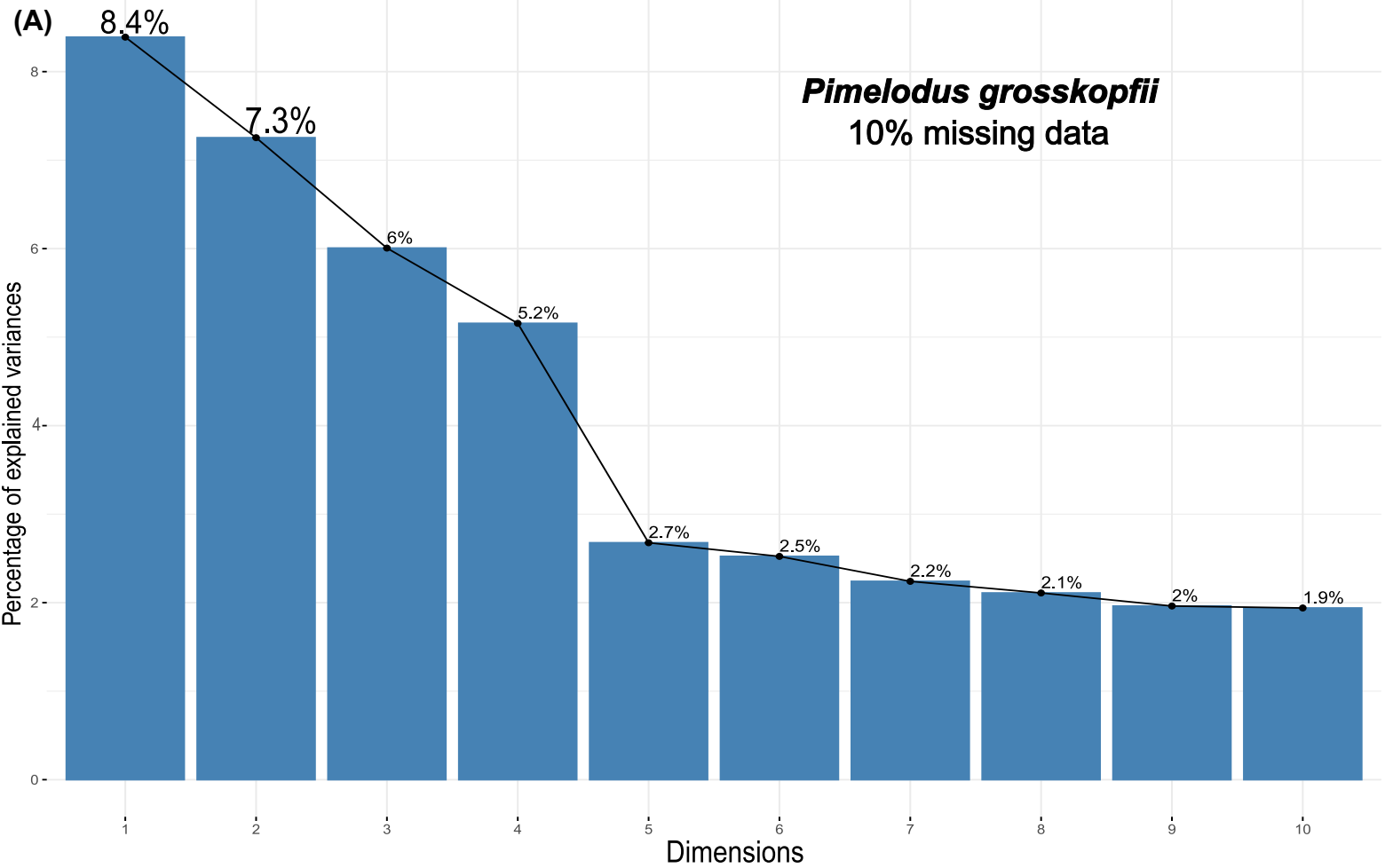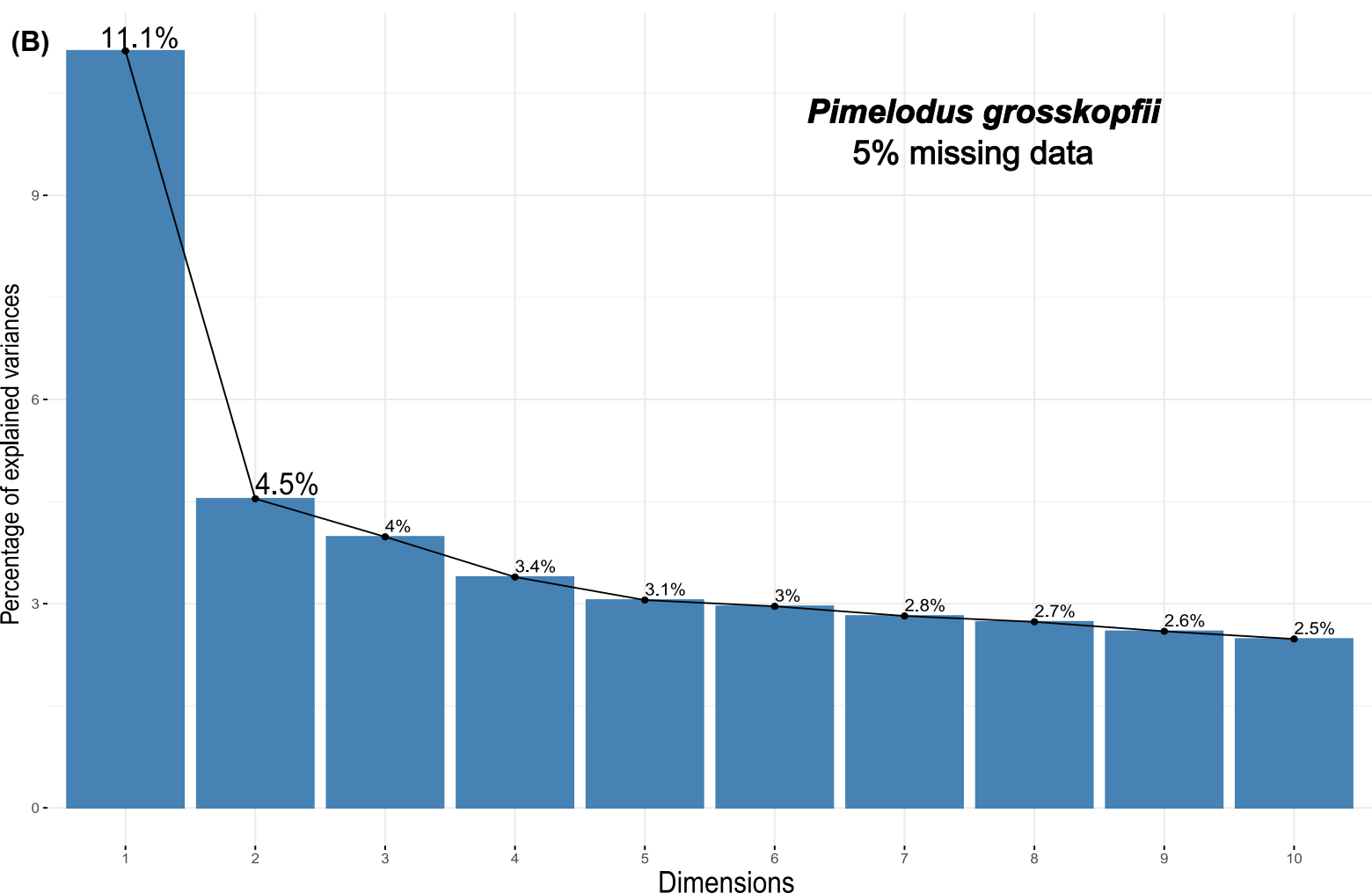

Supplement: S2 Fig — (PDF) [file pone.0351301.s002.pdf]

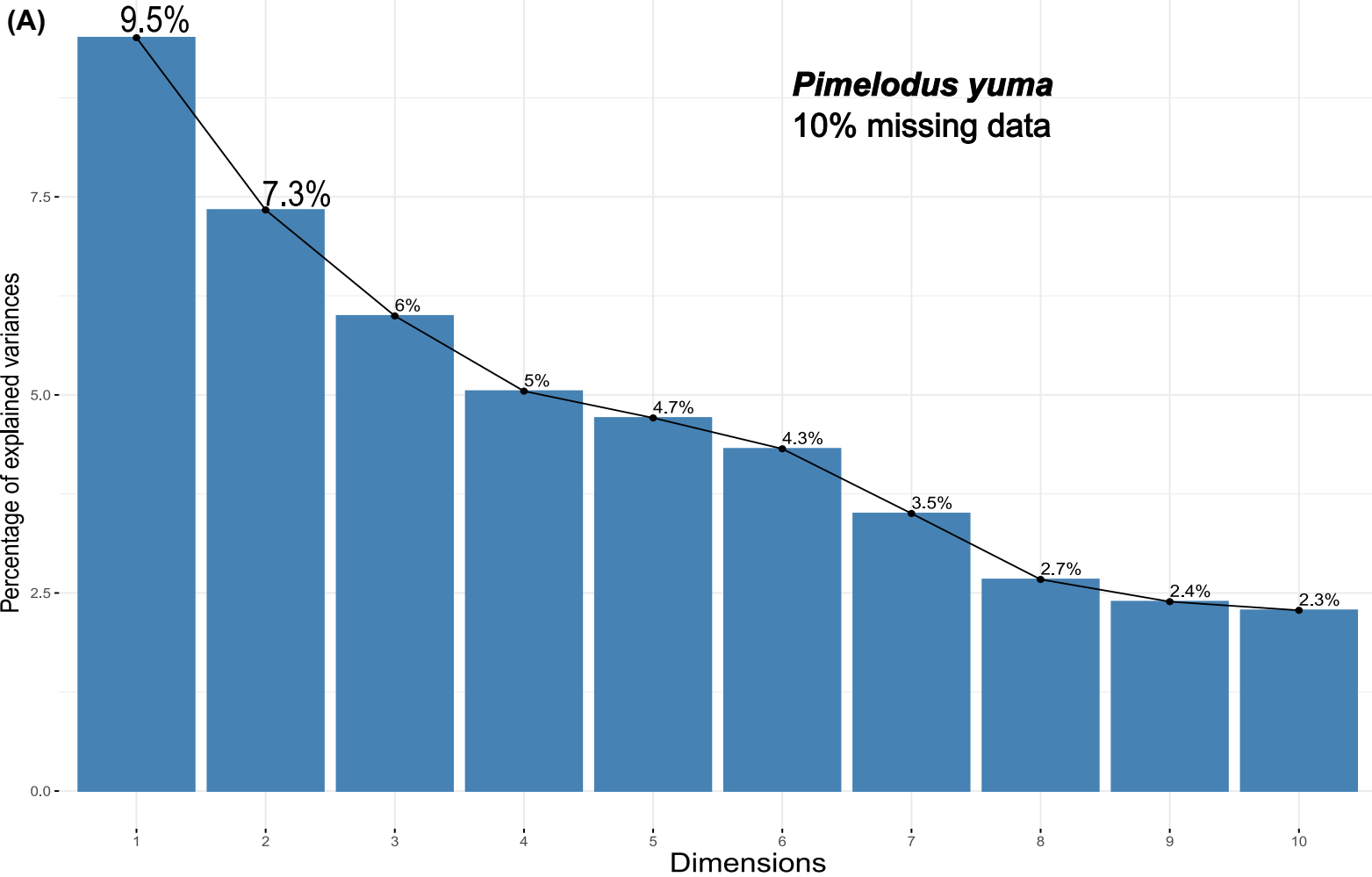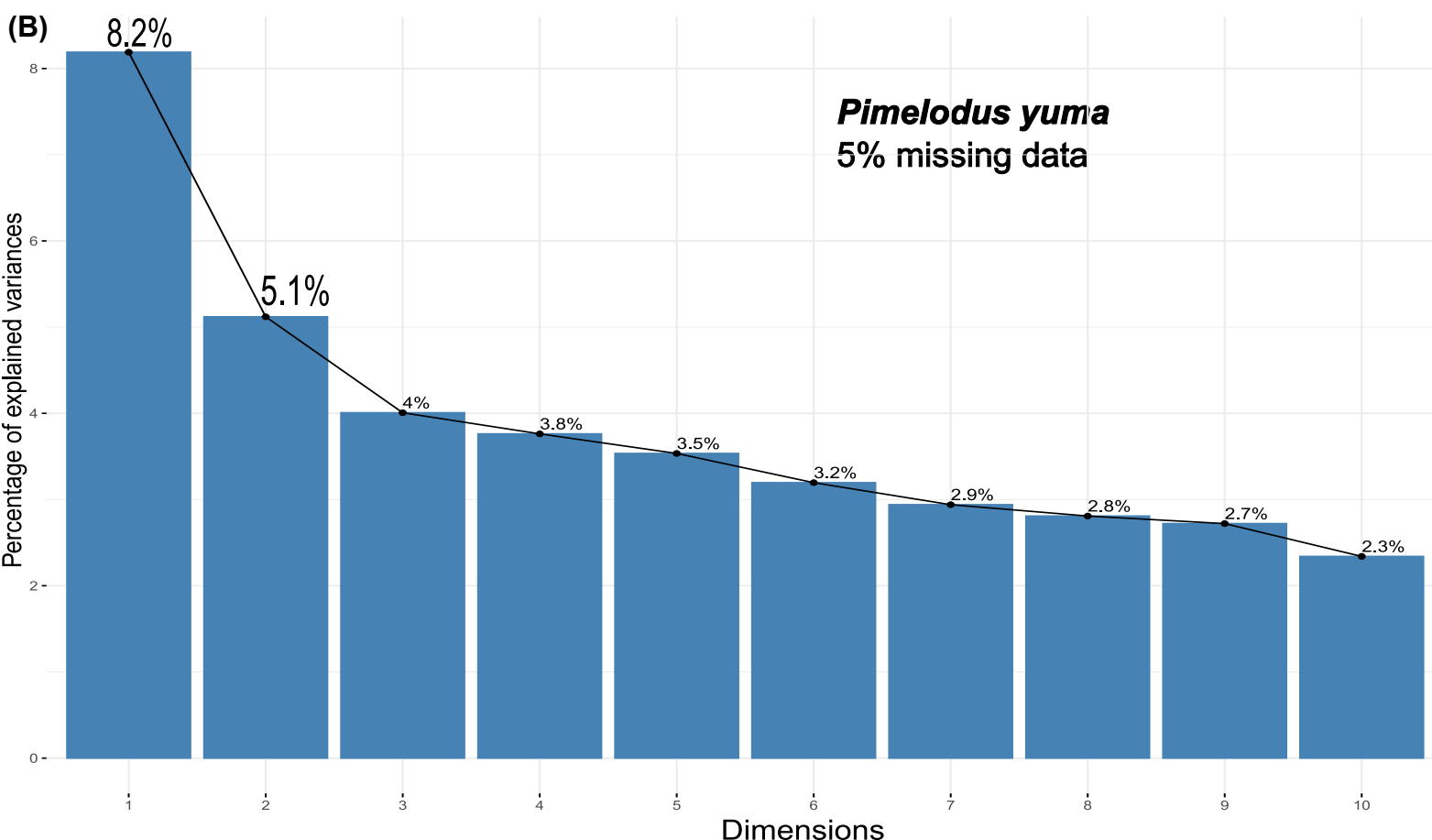

Supplement: S3 Fig — (PDF) [file pone.0351301.s003.pdf]

**MedMed K**

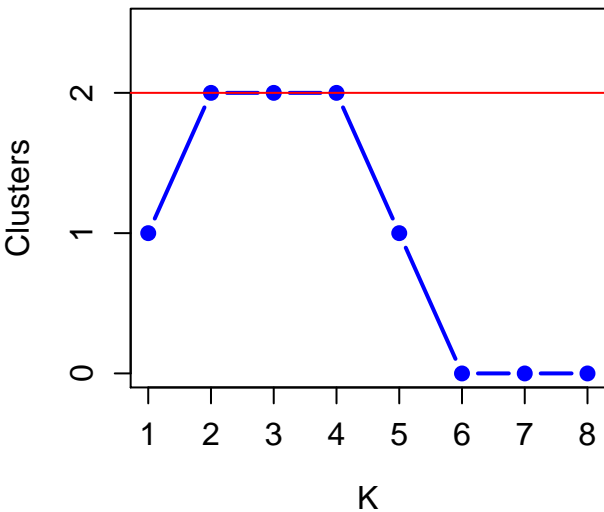

**MedMean K**

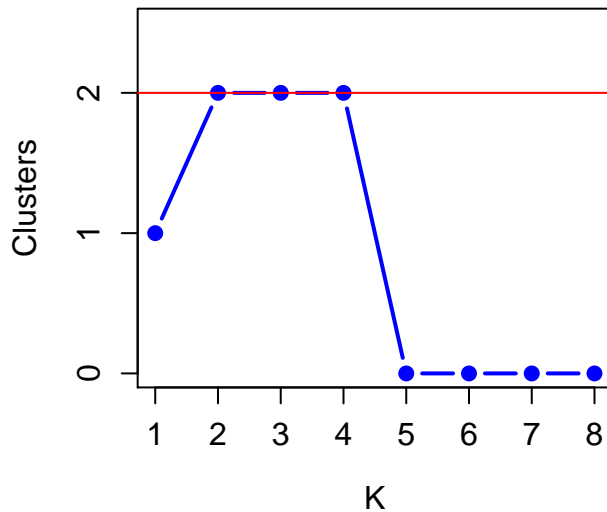

**MaxMed K**

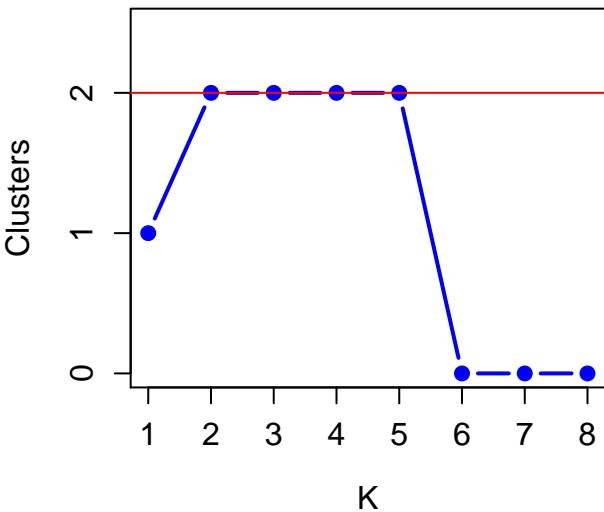

**MaxMean K**

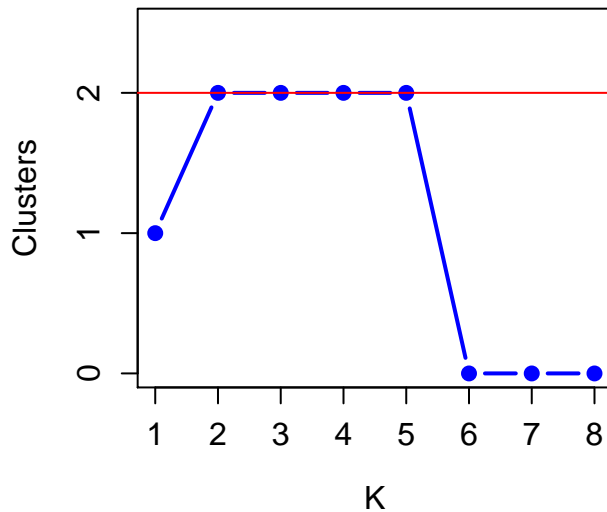

Supplement: S6 Fig — (PDF) [file pone.0351301.s006.pdf]

**MedMed K**

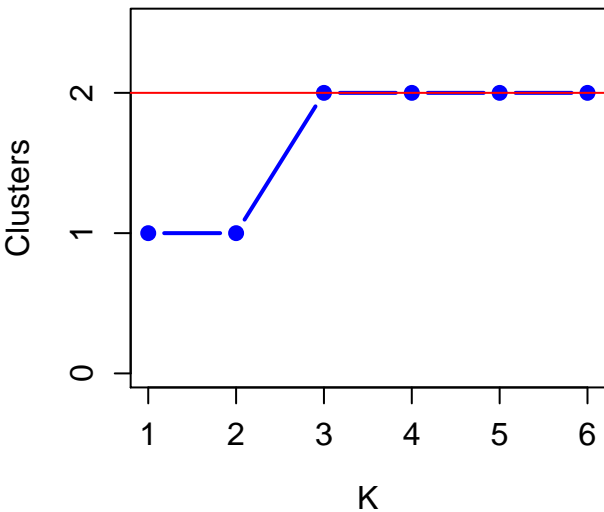

**MedMean K**

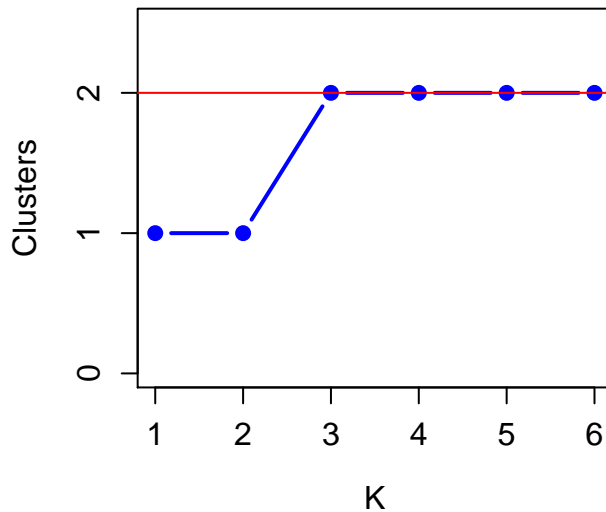

**MaxMed K**

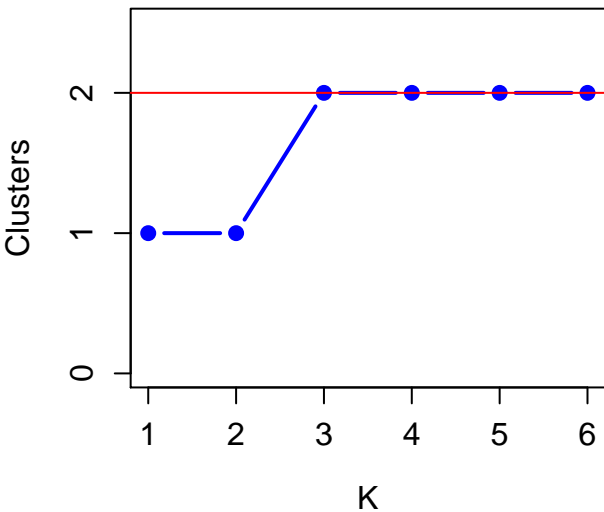

**MaxMean K**

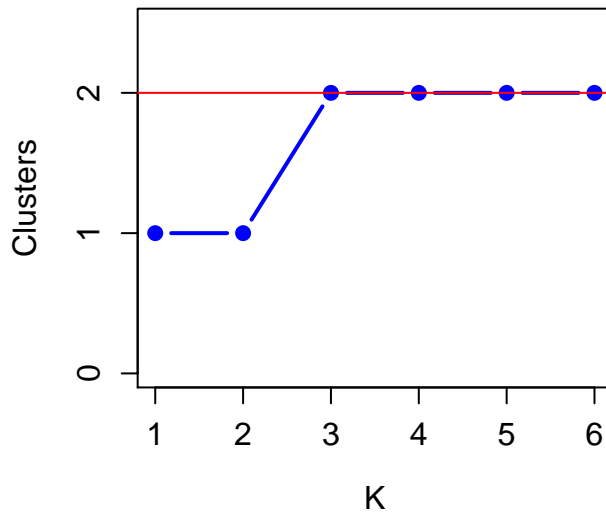

Supplement: S7 Fig — (PDF) [file pone.0351301.s007.pdf]

# GENOTYPIC FREQUENCY OF ADAPTATIVE LOCI BY STOCK

*Pimelodus grosskopfii*

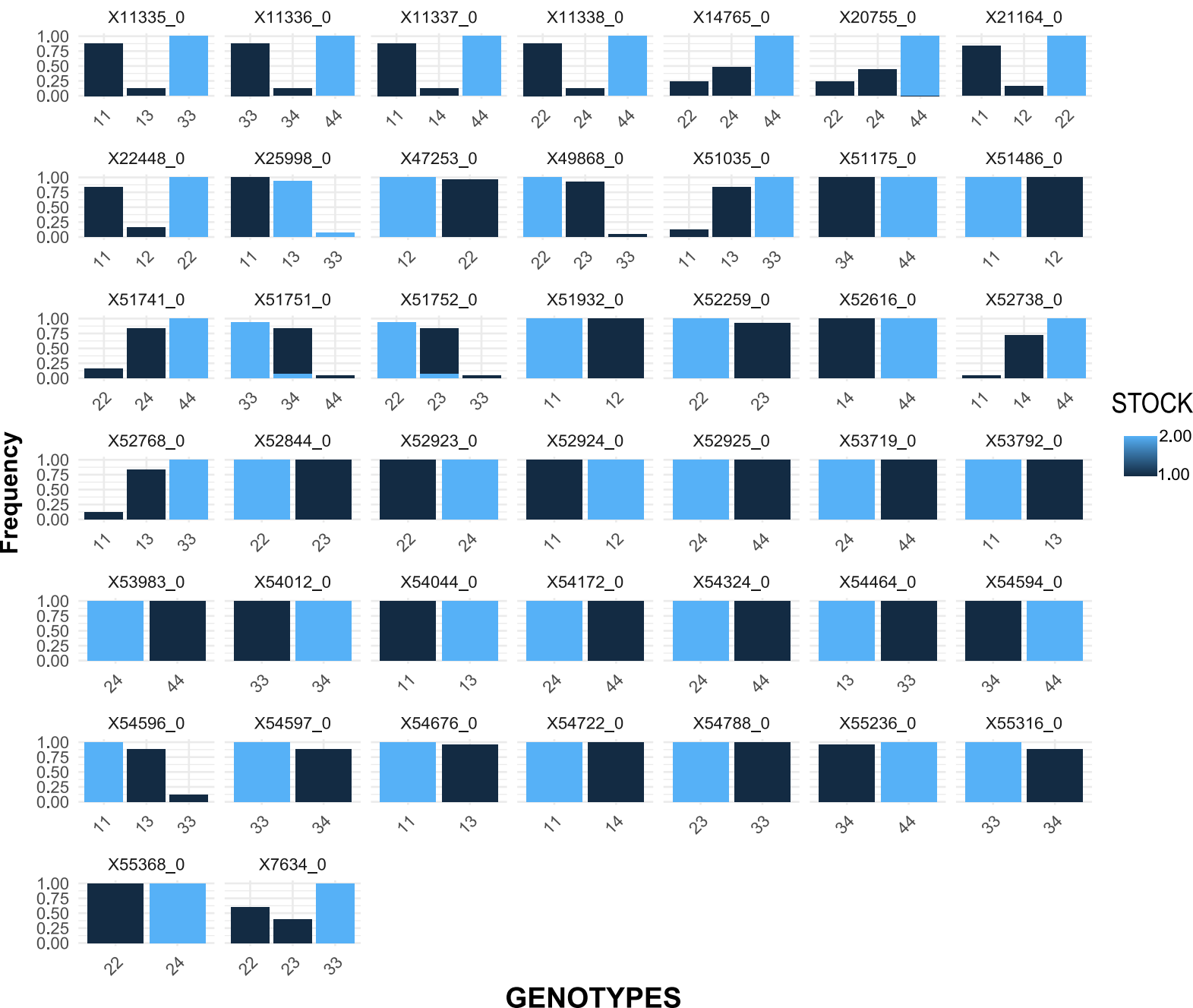

Supplement: S12 Fig — (PDF) [file pone.0351301.s012.pdf]

# GENOTYPIC FREQUENCY OF ADAPTATIVE LOCI BY STOCK

*Pimelodus yuma*

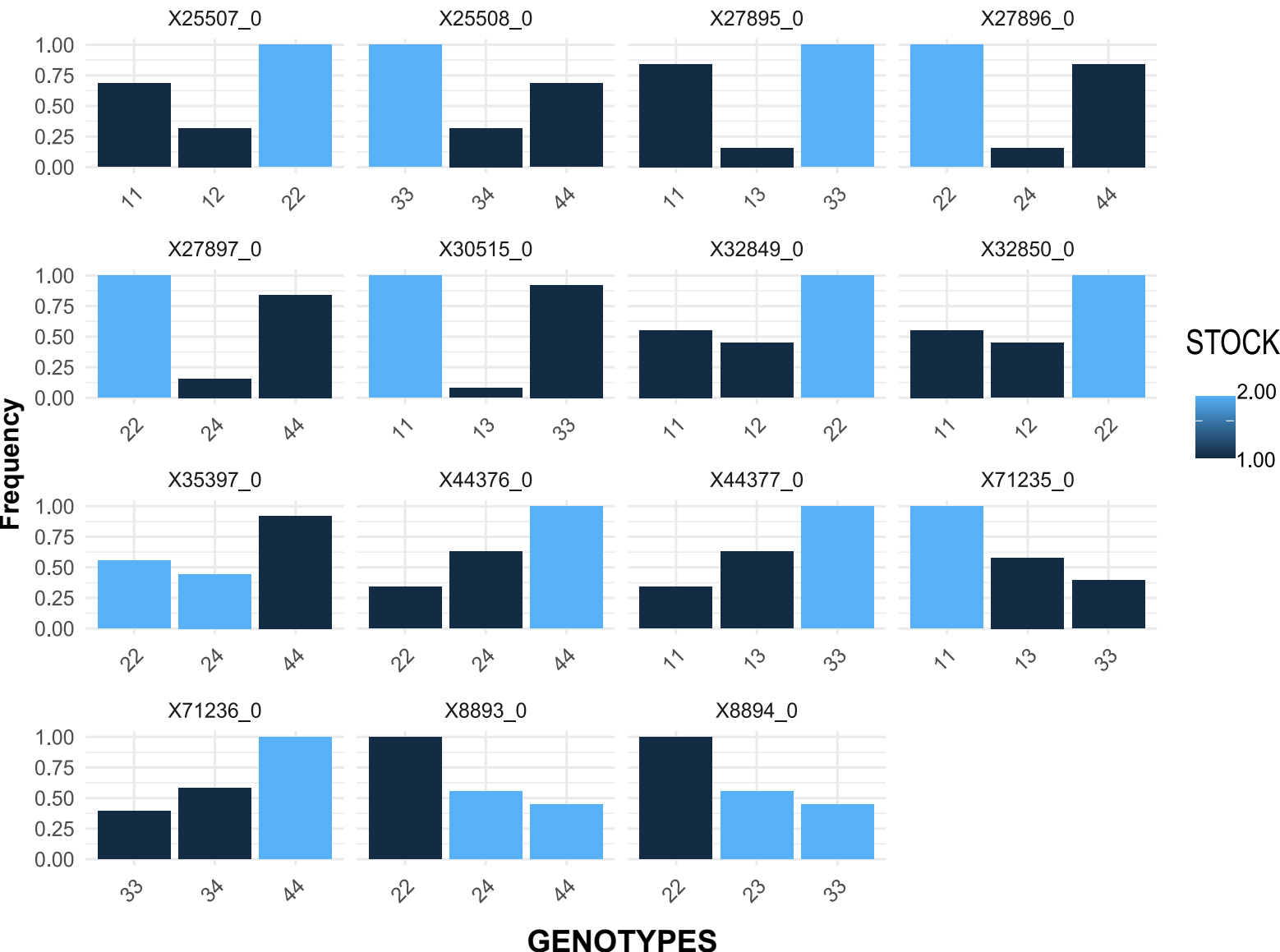

Supplement: S13 Fig — (PDF) [file pone.0351301.s013.pdf]
