# Supplementary material for: Genomic insights into population structure and adaptive variation of Pimelodus yuma and Pimelodus grosskopfii in the Magdalena-Cauca Basin
Source: PLoS One. 2026 Jun 5;21(6):e0351301. doi: 10.1371/journal.pone.0351301 (PMC13240932; doi:10.1371/journal.pone.0351301)
Supplement: S11 Fig — (PDF) [file pone.0351301.s011.pdf]

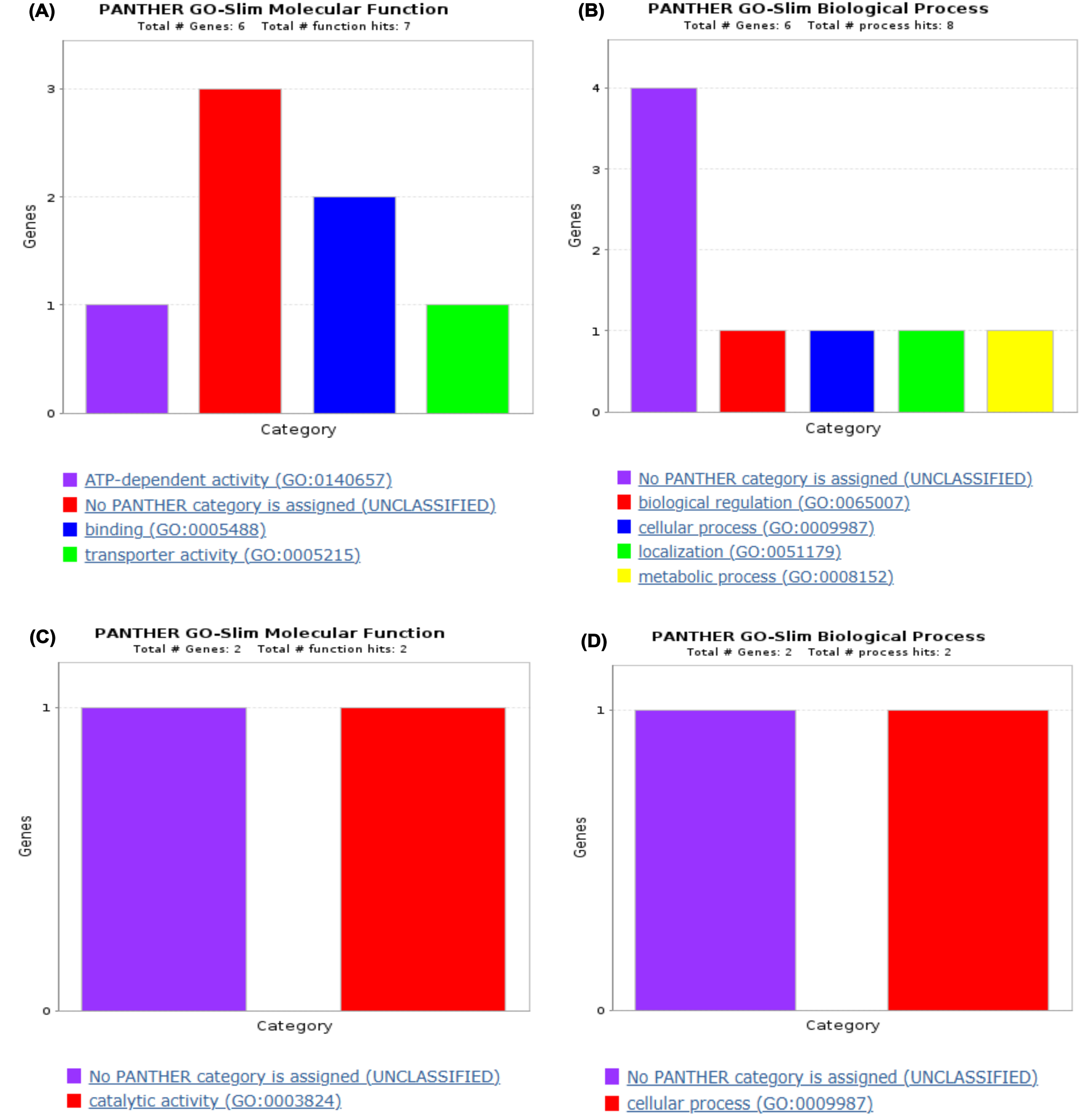
(A)
PANTHER GO-Slim Molecular Function
Total # Genes: 6 Total # function hits: 7
Genes
Category
ATP-dependent activity (GO:0140657)
No PANTHER category is assigned (UNCLASSIFIED)
binding (GO:0005488)
transporter activity (GO:0005215)
(B)
PANTHER GO-Slim Biological Process
Total # Genes: 6 Total # process hits: 8
Genes
Category
No PANTHER category is assigned (UNCLASSIFIED)
biological regulation (GO:0065007)
cellular process (GO:0009987)
localization (GO:0051179)
metabolic process (GO:0008152)
(C)
PANTHER GO-Slim Molecular Function
Total # Genes: 2 Total # function hits: 2
Genes
Category
No PANTHER category is assigned (UNCLASSIFIED)
catalytic activity (GO:0003824)
(D)
PANTHER GO-Slim Biological Process
Total # Genes: 2 Total # process hits: 2
Genes
Category
No PANTHER category is assigned (UNCLASSIFIED)
cellular process (GO:0009987)
